# Distinct APC Subtypes Drive Spatially Segregated CD4^+^ and CD8^+^ T-Cell Effector Activity during Skin Infection with HSV-1

**DOI:** 10.1371/journal.ppat.1004303

**Published:** 2014-08-14

**Authors:** Bethany L. Macleod, Sammy Bedoui, Jyh Liang Hor, Scott N. Mueller, Tiffany A. Russell, Natasha A. Hollett, William R. Heath, David C. Tscharke, Andrew G. Brooks, Thomas Gebhardt

**Affiliations:** 1 Department of Microbiology and Immunology, The University of Melbourne at the Peter Doherty Institute for Infection and Immunity, Melbourne, Victoria, Australia; 2 Division of Biomedical Science and Biochemistry, Research School of Biology, The Australian National University, Canberra, Australian Capital Territory, Australia; University of Glasgow, United Kingdom

## Abstract

Efficient infection control requires potent T-cell responses at sites of pathogen replication. However, the regulation of T-cell effector function *in situ* remains poorly understood. Here, we show key differences in the regulation of effector activity between CD4^+^ and CD8^+^ T-cells during skin infection with HSV-1. IFN-γ-producing CD4^+^ T cells disseminated widely throughout the skin and draining lymph nodes (LN), clearly exceeding the epithelial distribution of infectious virus. By contrast, IFN-γ-producing CD8^+^ T cells were only found within the infected epidermal layer of the skin and associated hair follicles. Mechanistically, while various subsets of lymphoid- and skin-derived dendritic cells (DC) elicited IFN-γ production by CD4^+^ T cells, CD8^+^ T cells responded exclusively to infected epidermal cells directly presenting viral antigen. Notably, uninfected cross-presenting DCs from both skin and LNs failed to trigger IFN-γ production by CD8^+^ T-cells. Thus, we describe a previously unappreciated complexity in the regulation of CD4^+^ and CD8^+^ T-cell effector activity that is subset-specific, microanatomically distinct and involves largely non-overlapping types of antigen-presenting cells (APC).

## Author Summary

HSV-1 is a widely distributed pathogen causing a life-long latent infection associated with periodic bouts of reactivation and severe clinical complications. Adaptive immune responses encompassing CD4^+^ and CD8^+^ T-cell activities are key to both the clearance of infectious virus and the control of latent infection. However, precisely how such T-cell responses are regulated, particularly within acutely infected peripheral tissues, remains poorly understood. Using a mouse model of HSV-1 skin infection, we describe a complex regulation of T-cell responses at the site of acute infection. These responses were subset-specific and anatomically distinct, with CD4^+^ and CD8^+^ T-cell activities being directed to distinct anatomical compartments within the skin. While IFN-γ-producing CD4^+^ T cells were broadly distributed, including skin regions a considerable distance away from infected cells, CD8^+^ T-cell activity was strictly confined to directly infected epithelial compartments. This unexpected spatial segregation was a direct consequence of the involvement of largely non-overlapping types of antigen-presenting cells in driving CD4^+^ and CD8^+^ T-cell effector activity. Our results provide novel insights into the cellular regulation of T-cell immunity within peripheral tissues and have the potential to guide the development of T-cell subset-specific approaches for therapeutic and prophylactic intervention in antimicrobial immunity and autoimmunity.

## Introduction

Infection results in the priming of pathogen-specific T-cell responses in LNs draining the site of infection. Depending on the nature of the pathogen, this critical step in generating adaptive immunity involves the interaction of naive T cells with various types of migrating and LN-resident DCs [1], [2]. During skin infection with herpes simplex virus (HSV)-1, LN-resident CD8α^+^ DCs and skin-derived CD103^+^ DCs can activate naïve CD8^+^ T-cells, presumably through the cross-presentation pathway involving the acquisition of noninfectious antigen [1]–[4]. By contrast, all subsets of skin-derived migratory DCs, including epidermal Langerhans cells, dermal CD11b^+^ and dermal CD103^+^ DCs, in addition to LN-resident CD8α^+^ DCs acquire the ability to stimulate naive HSV-specific CD4^+^ T cells [1], [2], [4]. Following appropriate activation by DCs, T cells undergo a program of clonal expansion, which is accompanied by the acquisition of effector functions and the induction of migration molecules that facilitate their infiltration of infected tissues.

While CD4^+^ helper T cells support the generation of antibody and CD8^+^ T-cell responses in lymphoid tissues, both CD4^+^ and CD8^+^ T-cells also contribute directly to pathogen control at sites of infection [5], [6]. The latter is achieved through two principle effector functions: the contact-dependent elimination of infected tissue cells and the local production of inflammatory and antimicrobial cytokines [5], [6]. The extent to which these T-cell activities contribute to immunity depends on the nature of the infection. For instance, control of non-cytopathic viruses, such as lymphocytic choriomeningitis virus, strictly requires cytolytic T-cell activity [7]. By contrast, immunity against cytolytic viruses, such as vaccinia and vesicular stomatitis virus, does not rely on target cell elimination by T cells [8]. Instead, under circumstances where infection will ultimately result in lytic cell death regardless of T-cell killing, pathogen containment and clearance is dependent on the production of cytokines by effector CD4^+^ and CD8^+^ T cells [9]–[11]. Together these diverse effector T-cell (T_EFF_) activities are essential for efficient immune protection, however, they may also cause the destruction of uninfected tissues, as seen in the context of immunopathology, autoimmunity or transplant rejection. Therefore, a detailed understanding of T-cell-mediated immunity in peripheral tissues forms an essential basis for therapeutic interventions to modulate T-cell responses against both harmful and innocuous antigens. Nevertheless, the cellular and molecular mechanisms controlling T-cell effector activities in nonlymphoid organs remain poorly defined [2], [10].

At its simplest, T-cell effector functions are regulated by T-cell receptor (TCR) stimulation through peptide-MHC complexes on APCs. Importantly in this respect, disengagement of the TCR from antigen-MHC complexes results in the immediate cessation of T-cell cytokine production [10], [12]. This “on-off cycling” of effector activity provides a sophisticated level of antigen specificity and places important temporal and spatial constraints on T_EFF_-cell responses [10]. As a consequence, effector T cells circulating through the blood or uninfected tissues are thought to shutdown cytokine production and to regain this effector function only upon reencounter with antigen in infected tissues [10]. In addition, noncognate signals delivered through inflammatory mediators and costimulatory molecules, such as interleukin (IL)-18, IL-12, type I IFNs or CD80 and CD86, may also trigger or further modulate T-cell cytokine production and cytotoxic activity [13]–[16]. Thus, the presence of appropriate APCs providing antigen stimulation together with accessory signals is critical in regulating T-cell immunity *in situ* and targeting effector activities to pathogen-containing tissues [2], [17]. Indeed, various types of professional and nonprofessional APCs, including monocyte-derived inflammatory DCs, B cells, neutrophils and parenchymal cells, have been suggested to elicit T-cell effector functions within nonlymphoid tissues [15], [18]–[21]. Key aspects in this regulation, however, particularly those pertaining to the infection status of APCs and the role of distinct APC subtypes in driving CD4^+^ versus CD8^+^ T_EFF_-cell responses, remain poorly understood.

Here, we define the cellular interactions that control T_EFF_-cell activity during the course of skin infection with HSV-1. We focus our analysis on the production of IFN-γ, a central component of adaptive immune responses. IFN-γexerts proinflammatory and regulatory effects on a variety of target cells, including the stimulation of antimicrobial activity and the induction of MHC molecules and inflammatory chemokines [5], [6]. Protection from HSV infection strictly requires T_EFF_-cell activities, with both CD4^+^ and CD8^+^ T cells contributing to virus control in skin, mucosa and sensory ganglia [22]–[26]. Moreover, efficient immunity against HSV infection requires IFN-γ [27] and, interestingly, it has been proposed that production of this cytokine rather than cytolytic activity is the major CD8^+^ T-cell mechanism for virus control in neuronal tissues [28], [29] as well as during lytic infection in genital mucosa [26]. The shared role of IFN-γ as a key effector molecule produced by both CD4^+^ and CD8^+^ T cells allowed us to directly compare the regulation of these T_EFF_-cell subsets side by side. We further took advantage of the tropism of HSV-1 for epithelial tissues [30] to document a distinct anatomical distribution of IFN-producing T_EFF_-cell subsets in relation to the presence or absence of infectious virus in different microanatomical compartments. Importantly, this unexpected spatial segregation of T_EFF_-cell effector activity was a direct result of the involvement of largely non-overlapping subsets of professional and nonprofessional APCs in driving CD4^+^ and CD8^+^ T_EFF_-cell responses.

## Results

### Distribution of IFN-γ-producing T_EFF_ cells

**Figure 1:**
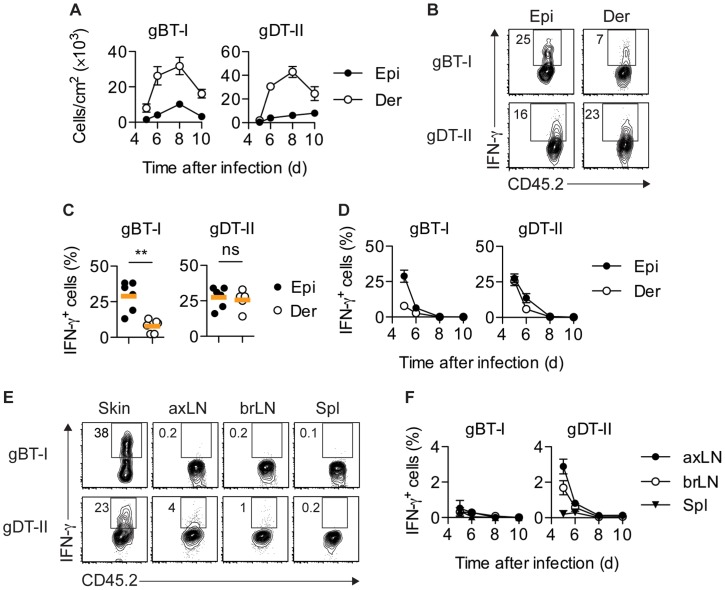
Distribution of IFN-γ^+^ T_EFF_ cells. Mice received naïve gBT-I or gDT-II cells prior to HSV-1 skin infection. (**A**) Quantification of T_EFF_ cells in epidermis (Epi) and dermis (Der) 5–10 days post-infection, tracked as CD45.2^+^CD45.1^+^ Vα2^+^ (gBT-I) or Vα3.2^+^ (gDT-II) cells (dispase digestion). Data pooled from *n* = 9–14 mice/time point. (**B**,**C**) Analysis of IFN-γ^+^ T_EFF_ cells in skin (dispase digestion) 5 days post-infection. **, *P*<0.01; ns, not significant by Mann Whitney test; *n* = 5–6 mice/group from 3–4 experiments. (**D**) Analysis of IFN-γ^+^ T_EFF_ cells 5–10 days post-infection; *n* = 4–7 mice/time point from 1–4 experiments. (**E**,**F**) Analysis of IFN-γ^+^ T_EFF_ cells in skin (gBT-I, epidermis; gDT-II, total skin), brachial (brLN) and axillary (axLN) LNs and spleen (5 days post-infection in **E**, or as indicated in **F**); *n* = 2–7 mice/organ/time point from 1–4 experiments.

To determine population kinetics and cytokine production by T_EFF_ cells in lymphoid and peripheral tissues, we utilized a skin infection with HSV-1 in combination with adoptive transfer of TCR-transgenic T cells specific for determinants derived from the HSV glycoproteins gB (CD8^+^ gBT-I cells) [31] and gD (CD4^+^ gDT-II cells) [4], respectively. Consistent with the tropism of HSV for epithelial tissues, immunofluorescence microscopy (IFM) of skin revealed that infection was largely confined to the epidermal layer and hair follicle epithelium (**Fig. S1A**). Separation of epidermal and dermal tissue (**Fig. S1B,C**) revealed that HSV-specific T_EFF_ cells began to infiltrate infected skin around 5 days post-infection, albeit with fewer cells in the smaller epidermal compartment (**Fig. 1A**). T_EFF_-cell numbers peaked around 8 days after inoculation and declined thereafter (**Fig. 1A**).

To analyze the production of IFN-γ by T_EFF_ cells *in situ*, we adopted protocols that facilitate intracellular cytokine staining following exposure to the Golgi inhibitor brefeldin A (BFA), either *in vivo* after intravenous injection [15], [32] and/or *ex vivo* immediately after tissue harvest and during enzymatic digestion [21]. Of note, in order to focus our analysis on IFN-γ production *in situ*, neither of these approaches involved overt restimulation with high concentrations of peptide antigen *ex vivo*. A considerable portion of gBT-I and gDT-II T_EFF_ cells in the epidermis and dermis of infected skin produced IFN-γ 5–6 days post-infection (**Fig. 1B**–**D**). Concomitant with clearance of infectious virus from skin [25], IFN-γ production by T_EFF_ cells ceased around day 7, with virtually no IFN-γ^+^ T_EFF_ cells present 8 days post-infection (**Fig. 1D**). Similar kinetics of IFN-γ production were also observed for endogenous CD8^+^ and CD4^+^ T_EFF_ cells (**Fig. S2A,B**). While roughly equal portions of gDT-II cells produced IFN-γ in the epidermal and dermal layers of skin 5 days after infection, the fraction of IFN-γ^+^ gBT-I cells was approximately 3-fold higher in the epidermis as compared to the dermis (**Fig. 1C,D**). Note, that dermal preparations contained hair follicles of epithelial origin and therefore also harbored some replicating virus (**Fig. S1A–C**). The broader distribution of IFN-γ^+^ gDT-II cells in the skin also extended to lymphoid tissues, with skin-draining axillary and brachial LNs, but not spleen, containing an appreciable fraction of IFN-γ-producing gDT-II cells (**Fig. 1E,F**). By contrast, IFN-γ^+^ gBT-I cells were virtually absent from all lymphoid tissues. Together, these results suggested a distinct anatomical distribution of IFN-γ-producing CD4^+^ and CD8^+^ T_EFF_ cells in both peripheral and lymphoid tissues.

### Confinement of IFN-γ^+^ CD8^+^ T_EFF_ cells to skin epithelium

**Figure 2:**
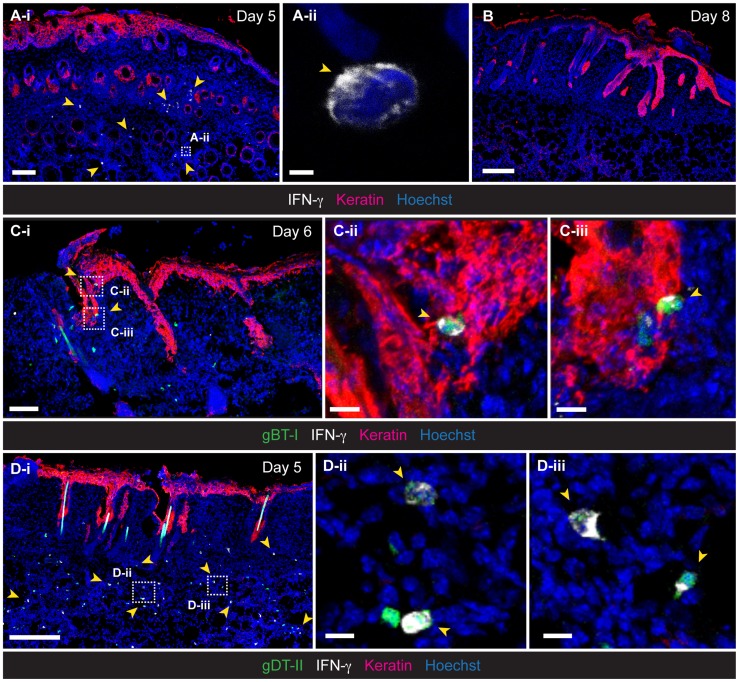
Localization of IFN-γ^+^ T_EFF_ cells in infected skin. IFM analysis of skin, 5 (**A**,**D**), 6 (**C**) and 8 (**B**) days post-infection, stained with anti-keratin and -IFN-γ antibodies, as indicated. (**C**,**D**) Detection of HSV-specific T_EFF_ cells after transfer of GFP^+^ naïve gBT-I (**C**) and gDT-II (**D**) cells prior to infection. Scale bars: **A-i**, 100 µm; **A-ii**, 2 µm; **B**, 200 µm; **C-i**, 70 µm; **C-ii**, 10 µm; **C-iii**, 10 µm; **D-i**, 200 µm; **D-ii**, 10 µm; **D-iii**, 10 µm. Photos representative of *n* = 4–6 mice/group.

To gain further insight into the microanatomical localization of IFN-γ-producing T_EFF_-cell subsets, we obtained skin tissue for IFM analysis. Staining of skin sections with anti-IFN-γ antibodies confirmed the presence of IFN-γ-producing cells during the acute phase of infection (**Fig. 2A,B**), with both endogenous CD4^+^ and CD8^+^ (**Fig. S2C,D**), as well as transgenic gBT-I and gDT-II T_EFF_ cells (**Fig. 2C,D**) contributing to this response. Interestingly, although gBT-I T_EFF_ cells were broadly distributed throughout the skin, IFN-γ^+^ gBT-I cells were strictly confined to the epidermis and hair follicle epithelium (**Fig. 2C**). By contrast, the majority of IFN-γ^+^ gDT-II cells localized to the dermal layer, where they were found either in association with hair follicles or in considerable distance to the epithelium (**Fig. 2D**). Thus, in contrast to the strict confinement of IFN-γ^+^ CD8^+^ T_EFF_ cells to the epithelium, the dermal layer was the predominant site of the CD4^+^ T-cell IFN-γ response.

### Antigen requirements for IFN-γ production by T_EFF_ cells

**Figure 3:**
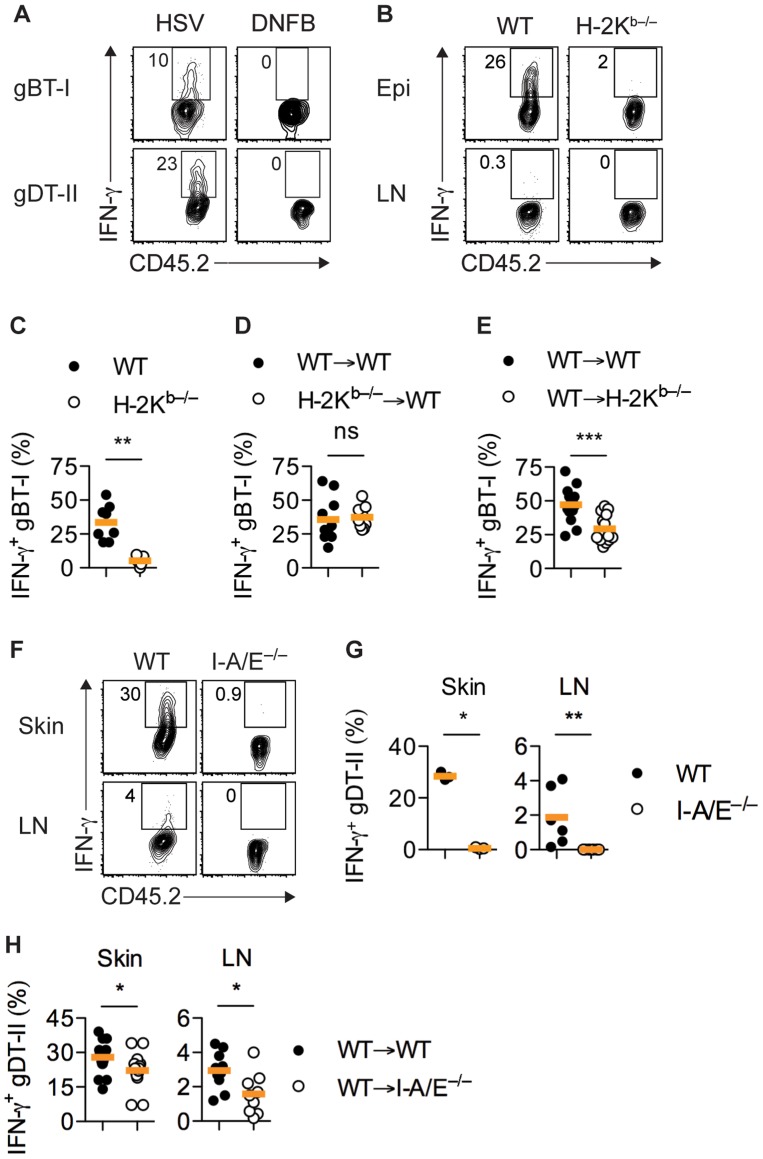
MHC-dependent activation of T_EFF_ cells by APCs. (**A**) Mice received naïve gBT-I or gDT-II cells prior to HSV-1 skin infection and DNFB treatment on opposite flanks. Analysis of IFN-γ^+^ gBT-I and gDT-II cells from skin (collagenase digestion) 5 days post-infection. Data representative of *n* = 2 experiments with skin pooled from 4 mice each. (**B**–**D**) Wild-type (WT) and H-2K^b−/−^ mice or WT→WT and H-2K^b−/−^→WT bone marrow chimeric mice were subjected to HSV-1 skin infection and transferred with *in vitro* activated gBT-I cells 3 days post-infection. (**E**) WT→WT and WT→H-2K^b−/−^ mice received naïve gBT-I cells prior to infection. (**B**–**E**) Analysis of IFN-γ^+^ gBT-I cells from axillary LN (**B**) and epidermis (Epi, dispase digestion, **C**–**E**) 5 days post-infection. **, *P*<0.01; ***, *P*<0.001; ns, not significant by Mann Whitney test; *n* = 7–14 mice/group from 3–5 experiments. (**F**,**G**) WT and I-A/E^−/−^ mice were subjected to HSV-1 skin infection and transferred with *in vitro* activated gDT-II cells 3 days later. Analysis of IFN-γ^+^ gDT-II cells from skin (collagenase digestion) and axillary LNs 5 days post-infection. *, *P*<0.05; **, *P*<0.01 by Mann Whitney test; 3 experiments with *n* = 2–4 mice/group; symbols for skin represent values from pooled tissues. (**H**) WT→WT and WT→I-A/E^−/−^ mice received naïve gDT-II cells prior to infection. Analysis of IFN-γ^+^ gDT-II cells in skin (collagenase digestion) and axillary LNs 5 days post-infection. *, *P*<0.05 by Mann Whitney test; *n* = 9–19 mice/group from 3 experiments.

The kinetics of IFN-γ production by T_EFF_ cells suggested that the presence of infectious virus was likely to play a role in the induction of cytokine production. This was indeed the case, as gBT-I and gDT-II T_EFF_ cells primed by HSV infection did not produce IFN-γ in non-specifically inflamed skin after treatment with 1-fluoro-2,4-dinitrobenzene (DNFB) (**Figs. 3A** **and S3A**). Likewise, *in vitro* activated gBT-I T_EFF_ cells transferred into HSV-infected mice lacking H-2K^b^ molecules (H-2K^b−/−^) did not produce significant amounts of IFN-γ in the skin (**Figs. 3B,C** **and S3B**). Transfer of activated T_EFF_ cells was necessary as H-2K^b−/−^ mice cannot prime naïve gBT-I cells due to lack of the relevant MHC-I restriction element. IFN-γ production by transferred gBT-I T_EFF_ cells was completely restored in similar experiments following HSV-1 infection of bone marrow chimeric mice in which H-2K^b^ molecules were expressed exclusively in radioresistant cells, but were absent from the radiosensitive hematopoietic compartment (**Figs. 3D** **and S3C**). By contrast, we observed a significant reduction in the frequency of *in vivo* primed IFN-γ^+^ gBT-I cells in chimeric mice in which H-2K^b^ molecules were selectively missing from radioresistant cells, when compared with fully MHC-I-sufficient control chimeras (**Figs. 3E** **and S3D**). Interestingly, the overall frequencies of IFN-γ^+^ gBT-I T_EFF_ cells appeared to be increased in this particular experimental set-up, possibly related to altered immune activation thresholds in previously irradiated recipient mice. Regardless, these results indicated that presentation of viral antigens by radioresistant epithelial cells, such as keratinocytes, Langerhans cells [33] and dendritic epidermal T cells (DETC) [34], was necessary and sufficient for optimal IFN-γ responses by gBT-I T_EFF_ cells.

IFN-γ production by gDT-II cells was also a consequence of antigen recognition, as *in vitro* activated gDT-II T_EFF_ cells transferred into infected MHC-II-deficient mice (I-A/E^−/−^) failed to produce IFN-γ (**Figs. 3F,G** **and S3E**). Transfer of activated gDT-II cells was necessary to overcome the inability of I-A/E^−/−^ mice to support CD4^+^ T-cell priming. By contrast, gDT-II T_EFF_ cells primed *in vivo* produced IFN-γ in chimeric mice in which only radiosensitive, but not radioresistant cells expressed MHC-II molecules, although we observed a moderate, yet significant reduction in the frequency of IFN-γ^+^ gDT-II cells in this situation (**Figs. 3H** **and S3F**). These results implied that bone marrow-derived MHC-II^+^ APCs were largely responsible for presentation of viral antigens and eliciting cytokine production by CD4^+^ T_EFF_ cells.

### DCs trigger IFN-γ production by CD4^+^ T_EFF_ cells

**Figure 4:**
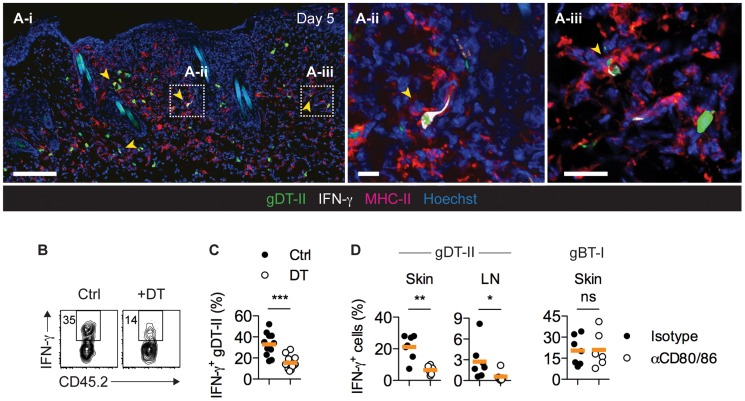
CD11c^+^ DCs drive IFN-γ production by CD4^+^ T_EFF_ cells *in vivo*. (**A**) Mice received GFP^+^ gDT-II cells prior to HSV-1 skin infection. IFM analysis of skin 5 days post-infection after staining with anti-MHC-II and -IFN-γ antibodies. Arrows indicate IFN-γ^+^ gDT-II cells. Scale bars: **A-i**, 100 µm; **A-ii**, 20 µm; **A-iii**, 20 µm. (**B**,**C**) CD11c.DTR mice received naïve gDT-II cells prior to HSV-1 skin infection and 4 days post-infection were treated with diphtheria toxin (DT) or PBS (Ctrl). Analysis of IFN-γ^+^ gDT-II cells in skin (collagenase digestion). ***, *P*<0.001 by Mann Whitney test; *n* = 11–15 mice/group from 3 experiments. (**D**) Wild-type mice received naïve gDT-II or gBT-II cells prior to infection and 4 days post-infection were treated with anti-CD80/86 (αCD80/86) or isotype control (Isotype) antibodies. Analysis of IFN-γ^+^ gDT-II cells in skin (collagenase digestion) and axillary LNs, and of IFN-γ^+^ gBT-I cells in epidermal sheets (dispase digestion) days 5 post-infection. *, *P*<0.05; **, *P*<0.01; ns, not significant by Mann Whitney test; *n* = 6–7 mice/group from 2–3 experiments.

Consistent with an involvement of MHC-II-expressing APCs in driving CD4^+^ T_EFF_-cell responses, MHC-II^hi^ cells accumulated in the skin during the first week post-infection [35] (**Fig. S4A,B**). The majority of these cells had a CD11c^int^CD11b^+^ phenotype and further expressed CD64 and MAR-1, identifying them as monocyte-derived inflammatory DCs [36], [37] (**Fig. S4C**). IFM of infected skin revealed a broad distribution of MHC-II^+^ cells, with the vast majority localizing to the dermal layer, where they were found in close proximity to IFN-γ^+^ gDT-II cells (**Fig. 4A**). Indeed, partial depletion of CD11c^+^ DCs upon diphtheria toxin (DT) treatment of CD11c.DTR mice resulted in a significant reduction in the frequency of IFN-γ^+^ gDT-II cells in infected skin (**Figs. 4B,C** **and S5A**). Furthermore, treatment of mice with antibodies blocking the costimulatory molecules CD80 and CD86, typically expressed by professional APCs such as DCs, also abrogated the CD4^+^ T_EFF_-cell IFN-γ in skin and draining LNs (**Figs. 4D** **and S5B**). In stark contrast, costimulation blockade had no bearing on IFN-γ production by gBT-I T_EFF_ cells (**Fig. 4D**). These experiments suggested that MHC-II^+^ DCs were the main drivers of cytokine production by CD4^+^ T_EFF_ cells. Nevertheless, in *Ccr2* ^−/−^ mice, an absence of monocyte-derived DCs [20], [38], which numerically dominated the cutaneous DC network during acute infection, rather increased than decreased the frequency of IFN-γ^+^ gDT-II and gBT-I cells (**Fig. S5C**,**D**), potentially related to impaired virus control in these mice [20]. Furthermore, we observed normal IFN-γ production by gDT-II and gBT-I T_EFF_ cells in the absence of Langerhans cells, CD103^+^ dermal DC and CD8^+^ LN-resident DCs upon DT treatment of Langerin.DTR mice [39], [40], and similarly, also in B-cell-deficient μMT mice [41] (**Fig. S5E**–**G**). These results suggested a level of redundancy regarding the involvement of different types of professional APCs in regulating CD4^+^ T_EFF_ activities in infected skin.

### Multiple DC subsets activate CD4^+^ T_EFF_ cells

**Figure 5:**
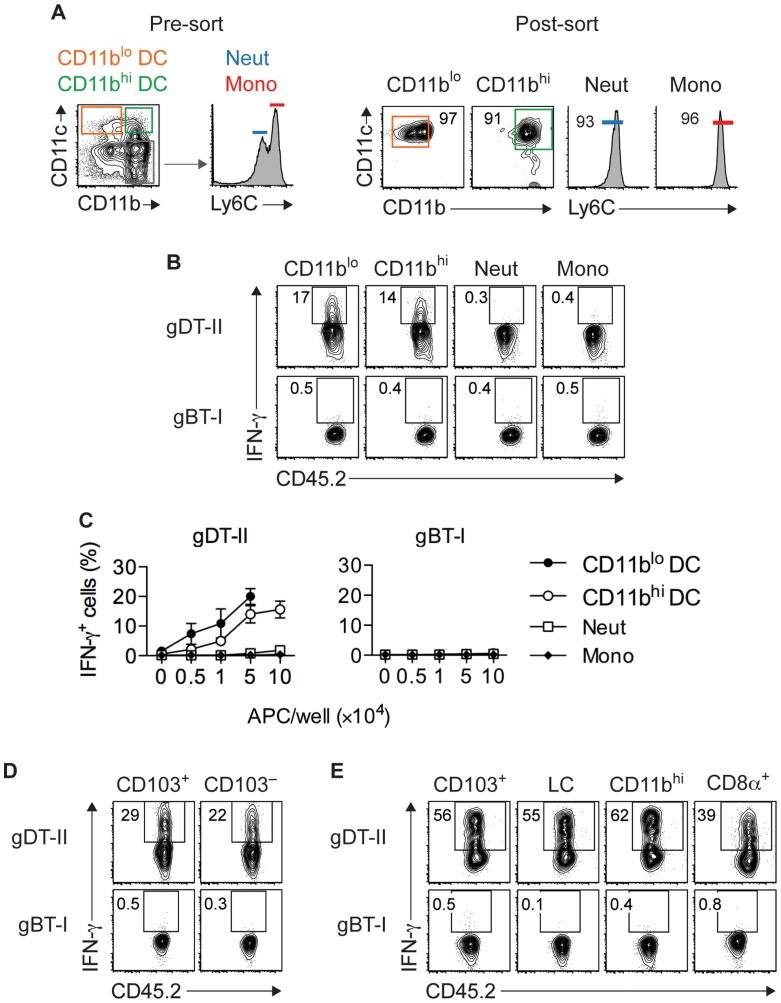
Multiple DC subsets elicit IFN-γ production by CD4^+^ but not CD8^+^ T_EFF_ cells. (**A**) Sorting strategy used to isolate CD11c^+^CD11b^lo^ and CD11c^+^CD11b^hi^ DCs, CD11c^−^Ly6C^int^ neutrophils (Neut) and CD11c^−^Ly6C^hi^ monocytes (Mono) from skin 5 days after HSV-1 infection. (**B**,**C**) Analysis of IFN-γ^+^ cells after culture of *in vitro* activated gDT-II and gBT-II effector cells with 5×10^4^ (**B**) or increasing numbers (**C**) of sorted APCs pooled from 10–20 mice. Culture period, 5 h for gBT-I and 18 h for gDT-II cells. Data pooled from 2–3 experiments. (**D**,**E**) Analysis of IFN-γ^+^ T_EFF_ cells cultured with indicated DC subsets pooled from 10–20 mice and sorted from skin (**D**; 1.5×10^4^) or axillary LNs (**E**; 5×10^4^) 5 days after infection as in **B**,**C**. LC, Langerhans cells. Representative plots from 2–3 independent experiments.

To more directly establish a role for DCs in CD4^+^ T-cell responses, we utilized an *ex vivo* stimulation assay in which APCs purified from infected mice were cocultured with *in vitro* generated T_EFF_ cells. Initial experiments revealed that maximal IFN-γ production occurred 18 hours after antigen-dependent restimulation for gDT-II and 5 hours for gBT-I T_EFF_ cells (**Fig. S6A**,**B**). We purified CD11c^hi^ DCs from HSV-infected skin and divided them into CD11b^hi^ and CD11b^lo^ subsets (**Fig. 5A**), with the former expected to contain monocyte-derived and dermal DCs and the latter expected to contain Langerhans cells and CD103^+^ dermal DCs [4], [37], [40]. Notably, both subsets induced robust IFN-γ production by gDT-II T_EFF_ cells, whereas monocytes (CD11b^+^CD11c^−^Ly6C^hi^) and neutrophils (CD11b^+^CD11c^−^Ly6C^int^) failed to do so (**Fig. 5B,C**). Unexpectedly, none of these APCs induced IFN-γ production by gBT-I T_EFF_ cells (**Fig. 5B,C**). This was not related to potentially compromised expression of H-2K^b^ molecules after APC isolation, since all subtypes triggered IFN-γ production by gBT-I T_EFF_ cells when pulsed with high doses of gB-peptide prior to cell sorting (**Fig. S6C**,**D**). In separate experiments, we specifically sorted CD103^+^ dermal DCs, as this DC subset is capable of cross-presenting viral antigens to CD8^+^ T cells during skin infection [4]. Once again, while both CD103^+^ and CD103^−^ CD11c^hi^ DCs triggered IFN-γ production by gDT-II T_EFF_ cells, neither of the two subsets activated gBT-I T_EFF_ cells (**Fig. 5D**). Remarkably, this disparate response also extended to DCs isolated from LNs draining the site of infection with CD103^+^, CD11b^+^, CD8α^+^ and Langerhans cell-containing CD103^−^CD11b^−^CD8α^−^ subsets inducing IFN-γ production by gDT-II, but not gBT-I T_EFF_ cells (**Fig. 5E**). Of note, gBT-I T_EFF_ cell unresponsiveness towards DC stimulation was observed irrespective of the culture period for 5–18 hours (not shown). Together, these results demonstrated that various skin-derived and LN-resident DC subsets acquired and presented viral antigen for the activation of CD4^+^ T_EFF_ cells. By contrast, none of these DCs elicited IFN-γ-production by CD8^+^ T_EFF_ cells, even though at least some of them, namely the CD8α^+^ and CD103^+^ subsets, have the ability to present viral antigens for the activation of naïve CD8^+^ T cells [3], [4].

### Epithelial APCs activate CD8^+^ T_EFF_ cells

**Figure 6:**
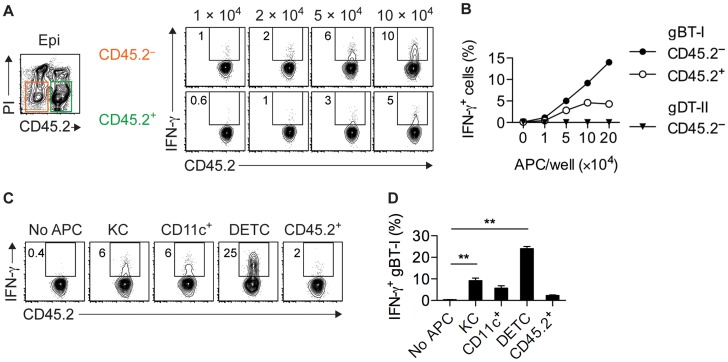
Epidermal APCs trigger IFN-γ production by CD8^+^ T_EFF_ cells. (**A**,**B**) Analysis of IFN-γ^+^ *in vitro* activated gBT-I (**A**,**B**) and gDT-II (**B**) T_EFF_ cells cultured (as in **Figure 5**) with increasing numbers of CD45.2^−^ and CD45.2^+^ APCs sorted from epidermal sheets (pooled from 20 mice) 4 days after HSV-1 skin infection. Data representative (**A**) or pooled from 2–3 experiments (**B**). (**C**,**D**) Analysis of IFN-γ^+^ *in vitro* activated gBT-I cells cultured with sorted APCs from epidermal sheets (pooled from 20 mice) 4 days after infection. APC numbers were 5×10^4^ for keratinocytes (KC), CD11c^+^ and residual CD45.2^+^CD11c^−^Vγ3^−^ (CD45.2^+^) cells; 3×10^4^ for DETCs. Data representative (**C**) or pooled from 2 experiments (**D**). **, *P*<0.01 by student t-test individually comparing specific APC subsets to control conditions (No APC).

Given that IFN-γ^+^ gBT-I cells were found exclusively in skin epithelium (**Figs. 1B–D** and **2B**), we reasoned that this compartment contained APCs capable of stimulating CD8^+^ T_EFF_ cells. Therefore, we purified CD45.2^+^ hematopoetic and CD45.2^−^ parenchymal and stromal cells from epidermal sheets of infected mice by cell sorting and tested their ability to activate CD8^+^ T_EFF_ cells. Note that here we used expression of CD45.2 to distinguish between hematopoeitc and non-hematopoeitc epidermal cells, whereas in other analyses we used this molecule as a marker for CD45.1^+^CD45.2^+^ gBT-I and gDT-II cells. Both fractions induced IFN-γ production by gBT-I T_EFF_ cells, although the keratinocyte-containing CD45.2^−^ subset appeared to be slightly more potent in this regard (**Fig. 6A,B**). Induction of IFN-γ production was antigen-specific, since co-cultured OT-I T_EFF_ cells of an irrelevant specificity did not respond to either of the APC subsets (**Fig. S7A**,**B**). Furthermore, stimulation of IFN-γ production by CD45.2^−^ epidermal APCs was specific for CD8^+^ T_EFF_ cells since these APCs failed to activate gDT-II T_EFF_ cells (**Figs. 6B** **and S7C**). In line with this, the vast majority of CD45.2^−^ epidermal cells from infected skin lacked expression of MHC-II molecules (**Fig. S7D**). To better define the nature of epidermal APCs capable of stimulating CD8^+^ T_EFF_ cells, we sorted these cells into keratinocytes (CD45.2^−^EpCAM^+^), DCs (CD45.2^+^CD11c^hi^), DETCs (CD45.2^+^Vγ3^+^), as well as residual CD45.2^+^CD11c^−^ cells. As expected, keratinocytes induced moderate levels of IFN-γ production by gBT-I T_EFF_ cells, and so did epidermal DCs (**Fig. 6C,D**), in contrast to their counterparts isolated from total skin preparations and LNs (**Fig. 5C**–**E**). While residual CD45.2^+^ epithelial cells had only a weak stimulatory capacity, remarkably, DETCs were by far the most potent APCs triggering IFN-γ production by gBT-I T_EFF_ cells (**Fig. 6C,D**).

### Only infected APCs activate CD8^+^ T_EFF_ cells

**Figure 7:**
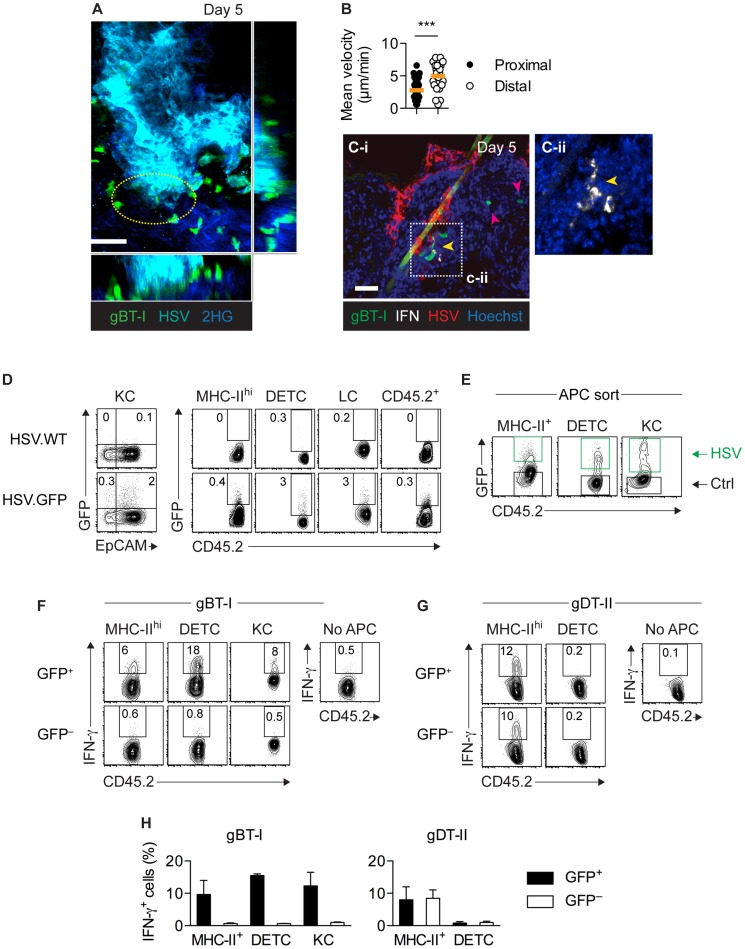
Only directly infected APCs activate CD8^+^ T_EFF_ cells. (**A**,**B**) Mice were subjected to skin infection with HSV.CFP and 3 days later received *in vitro* activated GFP^+^ gBT-I effector cells. Intravital two-photon microscopy of infected skin 5 days post-infection. (**A**) Maximum intensity projection image corresponding to **Movie S1**. Yellow circle indicates area with slow moving gBT-I cells. 2HG, second harmonic generation signal; scale bar, 50 µm. (**B**) Mean velocities of gBT-I cells in contact with (Proximal) or distal to (Distal) virus-infected cells from one movie, representative of 8 movies from 4 individual mice. (**C**) Mice received naïve GFP^+^ gBT-I cells prior to HSV-1 skin infection. IFM analysis of skin stained with anti-HSV-1 and -IFN-γ antibodies. Yellow arrow indicates IFN-γ^+^, red arrows IFN-γ^−^ gBT-I cells. Scale bar, 50 µm. Insert (**C-ii**) from indicated area in **C-i**, without green and red channels. Photos representative of *n* = 10 sections from 2 individual mice. (**D**–**H**) Mice were subjected to skin infection with HSV.WT or HSV.GFP. (**D**) Analysis of GFP^+^ keratinocytes (KC, CD45.2^−^EpCAM^+^), MHC-II^hi^ cells (CD45.2^+^MHC^hi^EpCAM^−^), DETCs (CD45.2^+^Vγ3^+^), Langerhans cells (LC, CD45.2^+^MHC-II^hi^EpCAM^+^) and residual CD45.2^+^ cells (MHC-II^−^Vγ3^−^) from epidermal sheets 5 days post-infection. Plots representative of *n* = 5 mice. (**E**) Sorting strategy used to purify epidermal GFP^+^ (HSV) and GFP^−^ (Ctrl) DETCs, MHC-II^hi^ cells and keratinocytes (pooled from 20 mice) 4 days post-infection. (**F–H**) Analysis of IFN-γ^+^ *in vitro* activated T_EFF_ cells cultured (as in **Figure 5**) with GFP^+^ or GFP^−^ MHC-II^hi^ cells (8–13×10^4^), DETCs (7–10×10^4^) and keratinocytes (14–16×10^4^) from epidermal sheets (pooled from 20 mice) 4 days after infection. Data representative (**F**,**G**) or pooled from 2–4 experiments (**H**).

Given that the epidermis was the predominant site of viral replication *in vivo*, we hypothesized that the stimulatory capacity of epidermal APCs resulted from their direct infection. In agreement, intravital two-photon microscopy of skin infected with a cyan fluorescent protein (CFP)-expressing HSV-1 strain revealed that slow-moving gBT-I T_EFF_ cells were swarming around virally infected epidermal cells (**Movie S1**). By contrast, gBT-I T_EFF_ cells more distal to infection foci displayed significantly higher mean velocities (**Fig. 7A,B**). Importantly, using IFM, we observed that IFN-γ^+^ gBT-I cells co-localized with HSV-infected cells in the epidermis and hair follicle epithelium (**Fig. 7C**). To further identify infected cells, we inoculated mice with a recombinant strain of HSV-1 expressing green fluorescent protein (HSV.GFP) and analyzed epidermal cells 5 days post-infection using flow cytometry. We observed small numbers of GFP-expressing cells amongst various populations of epidermal cells, including keratinocytes (CD45.2^−^EpCAM^+^), DETCs (CD45.2^+^Vγ3^+^), Langerhans cells (CD45.2^+^EpCAM^+^MHC-II^hi^), other DCs (CD45.2^+^EpCAM^−^MHC-II^hi^), as well as undefined CD45.2^+^MHC-II^−^ cells (**Fig. 7D**). As expected, GFP^+^ cells were absent after infection with the wild-type HSV-1 KOS strain. Next, we sorted GFP^+^ and GFP^−^ DETCs, MHC-II^hi^ DCs and keratinocytes from epidermal sheets (**Fig. 7E**) and tested their ability to stimulate T_EFF_ cells *ex vivo*. Strikingly, all GFP^+^, presumably infected, APCs were able to trigger IFN-γ production by gBT-I T_EFF_ cells (**Fig. 7F,H**). In contrast, IFN-γ production was not elicited by their GFP^−^ counterparts. Finally, DCs, but not DETCs, elicited IFN-γ production by gDT-II T_EFF_ cells, irrespective of their GFP-expression status (**Fig. 7G,H**). Together, these results indicated that various types of epidermal APCs induced cytokine production by CD8^+^ T_EFF_ cells. Importantly, direct viral infection was a strict requirement for their stimulatory capacity. Overall, our data highlight a previously unappreciated complexity in the regulation of T-cell effector activity that was subset-specific, microanatomically distinct and involved largely non-overlapping subsets of professional and nonprofessional APCs for CD4^+^ and CD8^+^ T-cell responses (**Fig. S8**).

## Discussion

Our results highlight a stringent and complex regulation of T_EFF_-cell responses that targets effector activities strictly to the site of infection and related lymphoid tissues. Thus, IFN-γ production was limited to the time of acute infection and occurred in an antigen-dependent fashion, requiring *in situ* restimulation via peptide-MHC complexes on bone marrow-derived professional APCs for CD4^+^ T_EFF_ cells and on directly infected tissue cells for CD8^+^ T_EFF_ cells. Consistent with a critical involvement of DCs in peripheral CD4^+^ T_EFF_-cell responses, we observed a pronounced accumulation of monocyte-derived inflammatory DCs in infected skin. Such DCs are thought to exert multiple functions, including the local production of inflammatory mediators [42], trafficking of antigen to lymph nodes [43] and replenishment of peripheral DC populations following the resolution of infection [35], [44]. In addition, our experiments employing genetic approaches, *ex vivo* stimulation assays and costimulation blockade provide compelling evidence that DCs in inflamed skin play a key role in stimulating CD4^+^ T-cell effector activity. These results reinforce the concept that DCs regulate various aspects of peripheral T-cell responses [2], [15], [20], [21], [45], [46]. In fact, the presence of DCs appeared to be essential for CD4^+^ T_EFF_-cell responses as non-professional APCs, such as keratinocytes and DETCs, largely lacked MHC-II expression and failed to elicit IFN-γ production in *ex vivo* assays. It is possible that certain APC subsets may dominate the regulation of peripheral CD4^+^ T_EFF_-cell activities, as suggested for CD11c^hi^CD11b^hi^ dermal DCs after skin injection of model antigens [21] or CCR2-depedent monocyte-derived inflammatory DCs during mucosal HSV-2 infection [20]. Nevertheless, our study revealed a considerable degree of redundancy in this regard, with various DC populations from skin and LNs displaying strong stimulatory capacities for CD4^+^ T_EFF_ cells. These results imply that all DC subsets, relative to their abundance in infected skin, contribute to CD4^+^ T_EFF_ activation *in vivo*. Consistent with this, we observed normal IFN-γ^+^ production in mice deficient in specific APC subsets, such as monocyte-derived DCs, Langerhans cells, CD103^+^ dermal DCs or B cells. According to our analysis, monocyte-derived inflammatory DCs are by far the most abundant DC subtype in HSV-infected skin [35] and therefore, may be the major drivers of peripheral CD4^+^ T_EFF_-cell responses during HSV-1 skin infection. Nevertheless, our results demonstrate that they may not be essential in this regard as other DC subsets may compensate for their absence.

The IFN-γ response by CD4^+^ T cells occurred in both draining LNs and the epithelial and dermal layers of infected skin, including regions a considerable distance away from infection foci in the epithelium. This remarkably broad distribution echoes the diverse functions of CD4^+^ T_EFF_ cells in infection control, ranging from the initiation of antibody class-switching in LNs to the regulation of inflammatory cell infiltration and activity as well as direct antimicrobial effects within infected tissues [5]. Interestingly in this respect, IFN-γ can exert long-range effects on target cells located as far as 80 µm from CD4^+^ T_EFF_-cell-APC conjugates, as recently shown for skin infection with *Leishmania major* [47]. Thus, DC-mediated CD4^+^ T_EFF_-cell activation in the dermis may be an essential component of the host defense that restricts infection to the skin epithelium and limits its spread after virus reemergence in sensory nerve endings. The importance of the CD4^+^ T_EFF_-cell response is further illustrated by the lack of CD8^+^ T_EFF_-cell IFN-γ production in the dermis, as shown here. Supporting this notion, CD4^+^ T_EFF_-cell responses are thought to dominate the clearance of HSV-1 from the skin [22], [23], most likely via antibody-independent functions such as direct inflammatory and antiviral activities [5], [48], [49].

In striking contrast to the stimulation requirements for CD4^+^ T_EFF_ cells, we identified nonprofessional APCs, such as keratinocytes and DETCs, as the main drivers of IFN-γ production by CD8^+^ T_EFF_ cells. According to our histological analysis, keratinocytes are the most abundant cell type in infected epidermis suggesting that they may be largely responsible for activating CD8^+^ T_EFF_ cells. In addition, various types of inflammatory cells that infiltrate the epithelial layer during infection may contribute to this response. Importantly, both keratinocytes and DETCs are highly susceptible to direct infection by HSV-1 *in vivo* [30], [50]. Indeed, their stimulatory capacity, and surprisingly also that of epidermal DCs, was strictly dependent on direct infection. DETCs are invariant γδ-T cells that form a dense network in the epidermis of mice and have been implicated in both innate and adaptive immune responses [51]. Interestingly, it has been speculated that human γδ-T cells may act as professional APCs capable of stimulating naïve CD4^+^ T cells [52], although we did not find evidence supporting a similar role for DETCs, as they failed to activate CD4^+^ T_EFF_ cells. Nevertheless, their contribution to CD8^+^ T-cell responses may be particularly relevant at lesion borders where high numbers of infected DETCs [50] could elicit strong IFN-γ responses required to curb the lateral spread of infection. In addition to DETCs, other epidermal-infiltrating T cells may also act as potent APCs for local CD8^+^ T_EFF_ cells upon infection with virus [53]. A role for infected T cells and DCs in triggering local CD8^+^ T_EFF_-cell activity is further supported by our observation that chimeric mice, in which only radiosensitive APCs could activate CD8^+^ T cells, had a residual IFN-γ response.

Despite the fact that all DC subsets could activate CD4^+^ T_EFF_-cells, only HSV-infected epidermal DCs were capable of activating CD8^+^ T_EFF_ cell to produce IFN-γ. Strikingly, uninfected DCs from the same location failed to stimulate CD8^+^ T_EFF_ cells, highlighting the importance of direct infection in determining the outcome of CD8^+^ T_EFF_-cell-DC interactions. Previous studies have suggested that various types of professional and nonprofessional APCs, including inflammatory DCs and neutrophils, can trigger IFN-γ production by CD8^+^ T_EFF_ cells during pulmonary infection with influenza virus [15], [18]. Given the ability of influenza virus to infect a broad range of target cells in addition to its primary tropism for lung epithelial cells [17], it is tempting to speculate that in this situation, direct infection may also be required for CD8^+^ T-cell activation. Supporting this notion, a large number of inflammatory DCs and neutrophils from influenza virus-infected lungs express viral antigens, most likely as a consequence of direct infection [15], [18], and infected neutrophils display a far superior ability to elicit CD8^+^ T_EFF_-cell cytokine production than their uninfected counterparts [18].

One of the more surprising findings from our study was that uninfected DCs failed to elicit IFN-γ production by CD8^+^ T_EFF_ cells, even though they had access to viral antigen and efficiently activated CD4^+^ T_EFF_ cells. We have previously shown that LN DCs are able to activate naïve CD8^+^ T cells at various stages during skin infection, unequivocally demonstrating that they present viral antigens in the context of MHC-I molecules [3], [4], [54]. The CD8α^+^ and CD103^+^ DC subsets are of particular interest in this regard, because those DCs cross-present viral antigens in draining LNs 5 days after HSV-1 infection [4], corresponding to the time point analyzed here. Despite this, CD103^+^ DCs from skin and LNs failed to trigger IFN-γ production by CD8^+^ T_EFF_ cells. This finding parallels observations with CD8^+^ T-cell responses to migratory DCs after influenza virus infection, where naïve but not memory T cells proliferate in response to antigen presented on migratory DCs [55].

Although not directly addressed in our study, it is tempting to speculate that the inability of uninfected DCs to activate CD8^+^ T_EFF_ cells may be a means to prevent their elimination by triggering cytotoxic effector functions. While T-cell killing of DCs has been proposed to represent a negative feedback regulation on T-cell priming [56], [57], it should be noted that prolonged antigen presentation is a common feature in a variety of infections [58]–[61]. Therefore, CD8^+^ T-cell-mediated elimination of DCs *in vivo* may be inefficient at best, which is also consistent with our preliminary data demonstrating the failure of gBT-I T_EFF_ cells to lyse CD103^+^ DCs from LN of HSV infected mice during short-term co-culture. The DC-dependent production of IFN-γ by CD4^+^ T_EFF_ cells observed in our study further supports this assumption. Of note in this respect, minute amounts of surface antigen can trigger T-cell cytotoxicity [62], [63]. Furthermore, cross-presenting nonprofessional APC, such as liver sinusoidal endothelial cells, can drive CD8^+^ T-cell TNFα production during viral hepatitis [64]. Thus, it appears unlikely that quantitative differences in antigen presentation between directly infected and cross-presenting DCs alone can explain the CD8^+^ T_EFF_-cell unresponsiveness described here. In the light of this, and given that infected epidermal DCs were indeed able to trigger CD8^+^ T_EFF_-cell IFN-γ production, we speculate that direct infection may alter the functional status of DCs, for instance through interference with putative inhibitory pathways [65], to allow for the activation of CD8^+^ T_EFF_ cells. Such modulation of DC stimulation thresholds may be particularly relevant when low levels of peptide-MHC-I complexes are presented to CD8^+^ T_EFF_ cells. Further studies will be required to elucidate the precise molecular mechanisms operating in DCs and/or T cells to prevent the activation of CD8^+^ T_EFF_ cells by uninfected cross-presenting DCs.

Overall, the stringent temporal, cellular and molecular constraints on T_EFF_-cell responses identified in our study are likely in place to prevent collateral damage and autoimmune inflammation initiated by T_EFF_-cell activation in tissues not involved in infection. Our results are compatible with a scenario where CD8^+^ T-cell responses are strictly focused on infected tissue compartments, whereas CD4^+^ responses may induce a more regional state of antimicrobial protection in tissues surrounding infection foci. Having identified dramatically distinct requirements for CD4^+^ and CD8^+^ T-cell effector activity, our study has provided novel insights into the regulation of cellular immune responses in nonlymphoid tissues. Such knowledge has the potential to guide the development of T-cell subset-specific approaches for therapeutic and prophylactic intervention in antimicrobial immunity and autoimmunity.

## Materials and Methods

### Ethics statement

All experiments were done according to Australian NHMRC guidelines contained within the Australian Code of Practice for the Care and Use of Animals for Scientific Purposes and under approvals ID1112038 and ID1112345 from the University of Melbourne Animal Ethics Committee.

### Mice

C57BL/6, B6.SJL-*PtprcaPep3b*/BoyJ (B6.CD45.1), gBT×B6.CD45.1 (gBT-I.CD45.1), gBT-I.EGFP, gBT-I.DsRed, OT-I×B6.CD45.1 (OT-I.CD45.1), gDT-II×B6.CD45.1 (gDT-II.CD45.1), gDT-II.EGFP, C57BL/6Ji-K^btm1^N12 (H-2K^b−/−^), B6.129S2-*H2^dlAb1-Ea^*/J (MHC-II^−/−^), CD11c.DTR, B6129.CCR2(*Ccr2* ^−/−^), Langerin.DTR.EGFP (Lg.DTR.EGFP) and μMT mice were bred in the Department of Microbiology and Immunology. gBT-I.CD45.1 and gDT-II.CD45.1 are F1 generation offspring expressing both CD45.1 and CD45.2.

### Viruses

HSV-1 KOS, KOS pCMV/EGFP Cre (HSV.GFP) and 17 HSVgDU_L_47ΔYFP (HSV.CFP) were grown and titrated as previously described [46]. HSV.GFP expresses an EGFP/Cre fusion gene under the control of the CMV-IE promoter from the intergenic space between UL3 and UL4 resulting in EGFP expression by infected cells. HSV.CFP was derived from HSV-1 gDU_L_47 (strain 17 expressing YFP from U_L_47 and CFP-tagged gD protein) [66], from which the YFP was removed and sequences from U_L_47 restored. HSV.GFP and HSV.CFP were made by homologous recombination between parent viral genomes with appropriate transfer plasmids in 293A cells after which green/yellow fluorescence were selected for and against, respectively. Final recombinants were verified by PCR and sequencing of relevant parts of the genome after at least three rounds of plaque purification.

### Bone marrow chimeric mice

Mice were irradiated with two of doses 550 cGy 3 hours apart followed by reconstitution with 5×10^6^ T-cell-depleted donor bone marrow cells and treatment with purified anti-Thy1 antibody (clone T24/31.7 from hybridoma supernatants) 1 day later. Chimeric mice were allowed to reconstitute for at least 8 weeks before experiments. WT→I-A/E^−/−^ chimeras were not treated with anti-Thy1 antibody and received 5×10^6^ enriched splenic CD4^+^ T cells from wild-type mice 1 day and 4 weeks after irradiation.

### Viral infections and DNFB, diphtheria toxin and antibody treatments

Mice were infected on their flanks with 1×10^6^ plaque-forming units of HSV-1, as previously described [25]. For DNFB treatment, 15 µL of 0.5% (w/v) DNFB was applied to flank skin, as previously described [67]. For depletion of CD11c^+^ cells, CD11c.DTR mice were injected with 200 ng diphtheria toxin (DT) or PBS intraperitoneally and intradermally 4 d post-infection. Langerin.DTR mice were injected with 500 ng DT or PBS control intraperitoneally. For costimulation blockade, mice were injected with 0.25 mg anti-CD80 (16-10A1) and -CD86 (GL1) blocking antibodies or rat IgG2 (B81-3) and IgG2a (R35-95) control antibodies (BD Pharmingen) intraperitoneally 4 d post-infection.

### Adoptive transfer of transgenic T cells

gBT-I and gDT-II cells were isolated from lymphoid tissues and gDT-II cells were further enriched by positive and negative selection using magnetic beads, as described previously [4]. 5×10^4^ gBT-I or 1×10^4^ gDT-II cells were transferred into naïve mice intravenously via the tail vein, respectively. For transfer of *in vitro* activated T cells, 1.5×10^6^ transgenic cells were injected.

### *In vitro* activation of transgenic T cells

Splenic gBT-I or OT-I cells were activated with peptide-pulsed splenocytes, as previously described [25]. Purified gDT-II cells were activated by co-culture for 5 days with 4×10^7^ irradiated wild-type splenocytes pulsed with 10 µM of gD_315–327_ in the presence of 2 µg LPS (Sigma). Cells were cultured in 20 ml RPMI 1640 (Department of Microbiology and Immunology) supplemented with 10% FCS (CSL), 5 mM HEPES (Gibco), 2 mM glutamine (Gibco), 5×10^−5^ M 2-β-mercaptoethanol (Sigma), antibiotics (Gibco, CSL) (RP-10) and 4 µg lipopolysaccharide. On days 2–4, respectively, all cultures were diluted 1∶2 in fresh medium containing 20 U/mL recombinant human IL-2 (PeproTech).

### Isolation of skin cells and *ex vivo* analysis of IFN-γ production

As indicated, skin tissue was chopped and digested with 3 mg/ml collagenase type 3 (Worthington Biochemicals, USA) and 5 µg DNase (Roche, Germany) for 90 min at 37°C. Alternatively, skin was digested in 2.5 mg/mL dispase II (Roche) diluted in PBS for 90 min at 37°C. Then, the epidermis and dermis were separated mechanically and epidermal sheets were incubated in trypsin/EDTA (0.25%/0.1%) (SAFC Biosciences), while the dermis was chopped and incubated in collagenase type 3 and DNase, as previously described [49]. When analyzing *ex vivo* IFN-γ production, 10 µg/mL Brefeldin A (Sigma) was included during each enzymatic digestion step. For some experiments, mice were additionally injected with 0.25 mg BFA intravenously 6 hours prior to sacrifice.

### Enrichment of DCs

Axillary lymph nodes of HSV-1-infected mice were desiccated with a scalpel blade and digested with continual mixing in RPMI 1640 containing 1 mg/mL collagenase type 3 and 2 µg/mL DNase for 20 min, prior to the addition of 600 µL 0.1 M EDTA and continual mixing for 5 minutes further. DCs were subsequently enriched for by magnetic beads, as previously described [4].

### *In vitro* cultures of T cells and APCs

APC subsets were stained with the appropriate monoclonal antibodies, purified by cell sorting using a FACSAria III (BD Pharmingen) and then washed and resuspended in RP-10. Increasing concentrations of the APCs were cultured with 1.25×10^4^ *in vitro* activated transgenic T cells in round bottom plates for 5 to 18 hours, as indicated, in the presence of 10 µg/mL BFA for the last 5 hours.

### Flow cytometry and antibodies

Antibodies were from BD Pharmingen: anti-CD3 (145-2C11), -CD4 (RM4-5), -CD8α (53-6.7), -CD11b (M1/70), -CD19 (ID3), -CD45.1 (A20), -CD80 (16-10A1), -CD86 (GL1), -IFN-γ (XMG1.2), -Ly6C (AL21), -NK1.1 (PK136), -Vα2 (B20.1) and -Vβ8 (MR5-2); from eBioscience: anti-CD45.2 (104) and -CD11c (N418); or from BioLegend: anti-CD326 (g8.8) and -Vα3.2 (RR2-16). For intracellular staining, cells were fixed with a Cytofix/Cytoperm kit (BD Pharmingen). A FACSCanto II (BD Pharmingen) and FlowJo software (TreeStar) were used for analysis. Propidium iodide (Sigma Aldrich) and SPHERO calibration particles (BD Pharmingen) were added for identification of live cells and enumeration.

### Immunofluorescence and confocal microscopy

Skin was fixed at room temperature for 30 min in PLP buffer (0.2 M NaH2PO4, 0.2 M Na2HPO4, 0.2 M L-lysine and 0.1 M sodium periodate with 2% paraformaldehyde), washed twice with PBS and incubated for 30 min in 20% sucrose, prior to being embedded, frozen, cut and stained as previously described [48]. IFN-γ staining (AlexaFluor647, BD Pharmingen) was performed overnight at 4°C (1∶75 in PBS containing 2.5% [w/v] donkey serum). Anti-keratin-5 and -14 polyclonal antibodies were from Jomar Biosciences; anti-CD4 (RM4-5) from BioLegend; anti-CD8 (53.67) from BD Pharmingen and polyclonal anti-HSV from Dako North America. Slides were mounted with ProLongGold antifade media (Invitrogen), air-dried and viewed using a Zeiss LSM700 confocal microscope and Imaris 7.1 software (Bitplane).

### Intravital two-photon microscopy

Mice were anesthetized and HSV-1-infected flank skin was mounted on an imaging platform and acquired with an upright LSM710 NLO multiphoton microscope as described previously [48]. Imaging data was processed and automatic cell tracking aided by manual corrections was performed with Imaris 7.1 software. For movies, image sequences were composed in Adobe After Effects CS5.

### Statistical analysis

Graphs were plotted using Prism 5 (Graphpad) and comparison of data sets was performed by one-way analysis of variance followed by Tukey post-test, or Mann-Whitney or student t tests, as indicated. All graphs depict means ± s.e.m..

## Supporting Information

Figure S1 **Topography of HSV-1 skin infection and preparation of epidermal sheets.** (**A**) IFM analysis of skin stained with anti-HSV antibody 5 days after infection. Arrows indicate HSV-infected epithelial cells in the epidermis (Epi) and hair follicles (HF). Scale bars: **A-i**, 100 µm; **A-ii**, 50 µm. (**B**) Schematic diagram and (**C**) IFM analysis depicting the enzymatic and mechanical separation of epidermis and dermis. Note that the dermis preparation contains hair follicles (HF) of epithelial origin. Epithelial cells depicted by staining with anti-keratin antibody. Scale bar, 200 µm.
(TIF)

Figure S2 **Distribution of endogenous IFN-γ^+^ T_EFF_ cells in infected skin.** (**A**,**B**) Mice were subjected to HSV-1 skin infection. Analysis of IFN-γ^+^ (**A**) CD8^+^ and (**B**) CD4^+^ T cells (collagenase digestion) 5 and 8 days post-infection. (**C**,**D**) IFM analysis of skin 5 days after infection stained with anti-HSV-1, -IFN-γ, -CD8 (**C**) or -CD4 (**D**) antibodies. Scale bars, **C-i**, 100 µm; **C-ii**, 20 µm; **D-i**, 100 µm, **D-ii**, 20 µm. Photos representative of *n*>5 sections from 2 mice/group.
(TIF)

Figure S3 **Experimental setups.** (**A**) Experimental setup for **Figure 3A**. Wild-type mice received naïve gBT-I or gDT-II cells and 1 day later were subjected to HSV-1 skin infection on their left flank followed by skin treatment with DNFB on the right flank 2 days post-infection. Analysis of IFN-γ^+^ gBT-I and gDT-II cells from both flanks 5 days post-infection. (**B**,**C**) Experimental setup for **Figure 3B**–**3D**. Wild-type (WT) and H-2K^b−/−^ mice (**B**), or WT→WT and H-2K^b−/−^→WT bone marrow chimeric (**C**) were subjected to HSV-1 skin infection and 3 days later received *in vitro* activated gBT-I effector cells. Analysis of IFN-γ^+^ gBT-I cells isolated from epidermal sheets 5 days post-infection. (**D**) Experimental setup for **Figure 3E**. WT→WT and WT→H-2K^b−/−^ mice received naïve gBT-I cells and 1 day later were subjected to infection. Analysis of IFN-γ^+^ gBT-I cells from epidermal sheets 5 days post-infection. (**E**) Experimental setup for **Figure 3F and 3G**. Wild-type (WT) and I-A/E^−/−^ mice were subjected to HSV-1 skin infection and 3 days later received *in vitro* activated gDT-II cells. Analysis of IFN-γ^+^ gDT-II cells isolated from skin and axillary LNs 5 days post-infection. (**F**) Experimental setup for **Figure 3H**. WT→WT and WT→I-A/E^−/−^ mice received naïve gDT-II cells and 1 day later were subjected to infection. Analysis of IFN-γ^+^ gDT-II cells from skin and axillary LNs 5 days post-infection.
(TIF)

Figure S4 **Infiltration of HSV-infected skin by CD11c^+^MHC-II^+^ APCs.** (**A**–**C**) Mice were subjected to HSV-1 skin infection. Analysis of APCs from skin (collagenase digestion) at the indicated time points. (**A**) Plots gated on PI^−^CD45.2^+^ cells. (**B**) Enumeration of CD11c^int/+^MHC-II^+^ DCs. (**C**) Analysis of CD11b, CD103, CD64 and MAR-1 expression on DC populations gated as indicated. Data from *n* = 4–5 mice/per group.
(TIF)

Figure S5 **T_EFF_-cell activation in absence of specific APC subtypes.** (**A**) Depletion of DCs in DT-treated CD11c.DTR mice. Representative plots, gated on CD45.2^+^PI^−^ cells, show the proportion of CD11c^+^MHC-II^+^ DCs in spleen and skin of CD11c.DTR mice treated with PBS or DT according to **Figures 4B and 4C**. (**B**) Experimental setup for **Figure 4D**. Wild-type mice received naïve gBT-I or gDT-II cells prior to HSV-1 skin infection and were treated with blocking anti-CD80 and -CD86 or isotype control antibodies 4 days post-infection. Analysis of IFN-γ^+^ gBT-I and gDT-II cells in epidermal sheets (gBT-I, dispase digestion) or skin (gDT-II, collagenase digestion) and axillary LNs 5 days post-infection. (**C**,**D**) Wild-type (WT) or *Ccr2* ^−/−^ mice received naïve gBT-I or gDT-II cells prior to HSV-1 skin infection. (**C**) Representative plots show the proportion of LyC^hi^Ly6G^−^ monocytes and of CD11c^+^MHC-II^hi^ APCs in the skin of WT and *Ccr*2^−/−^ mice 5 days post-infection. (**D**) Analysis of IFN-γ^+^ gBT-I (epidermis, dispase digestion) and gDT-II cells (dermis, collagenase digestion). *, *P*<0.05; ns, not significant by Mann Whitney test; *n* = 4–5 mice/group from 1 experiment (for gBT-I) and *n* = 11–16 mice/group from 4 experiments (for gDT-II). (**E**,**F**) Depletion of langerin^+^ cells in DT-treated Langerin.DTR mice. Langerin.DTR mice were subjected to HSV-1 skin infection and 4 days later treated with DT (500 ng) or PBS (Ctrl). Representative plots depict the proportion of Langerhans cells (MHC-II^hi^ EpCAM^+^) amongst PI^−^CD45.2^+^ cells in skin (collagenase digestion). (**F**) Mice received naïve gBT-I or gDT-II cells prior to infection. gBT-I-recipient mice were treated with DT (500 ng) or PBS (Ctrl) 4 days post-infection, and gDT-II-recipient mice were treated 2 days prior and 2 and 4 days after infection. Analysis of IFN-γ^+^ gBT-I cells in the epidermis (dispase digestion) or gDT-II cells in the skin (collagenase digestion). Ns, not significant by Mann Whitney test; *n* = 10–14 mice/group from 2–4 experiments. (**G**) Wild-type (WT) or μMT mice received naïve gDT-II cells prior to infection. Analysis of IFN-γ^+^ gDT-II cells in skin (collagenase digestion). Data from 2 experiments with skin tissue pooled from 4 individual mice each.
(TIF)

Figure S6 **T_EFF_-cell IFN-γ production triggered by peptide-pulsed APCs.** (**A**,**B**) Analysis of IFN-γ^+^ *in vitro* activated gBT-I and gDT-II effector cells cultured for 5–18 hours in the absence (Ctrl) or presence of gB_498–505_-peptide (gBT-I) or 1×10^5^ splenocytes and gD_315–237_-peptide (gDT-II). Representative plots gated on gBT-I and gDT-I cells, as indicated. (**C**,**D**) Mice were subjected to HSV-1 skin infection and 5 days post-infection APCs were isolated from skin (collagenase digestion), pulsed with 0.1 µg/mL gB_498–505_ peptide for 1 hour, and then sorted into CD11c^+^CD11b^lo^ and CD11c^+^CD11b^hi^ DCs, CD11c^−^Ly6C^int^ neutrophils (Neut) and CD11c^−^Ly6C^hi^ monocytes (Mono), as described in **Figure 5A**. (**C**,**D**) Analysis of IFN-γ^+^ *in vitro* activated gBT-I effector cells cultured for 5 hours in the presence of the indicated APC subsets (5×10^4^ each in **C**, increasing numbers as indicated in **D**). Data representative of (**C**) or pooled from (**D**) 2 experiments.
(TIF)

Figure S7 **Distinct epidermal APC subsets trigger IFN-γ production by CD4^+^ and CD8^+^ T_EFF_ cells.** (**A**,**B**) Analysis of IFN-γ^+^ *in vitro* activated gBT-I (Vβ8^+^) and OT-I (Vβ8^−^) cells co-cultured in the absence (**A**) or presence of increasing numbers of CD45.2^−^ or CD45.2^+^ cells (**B**) from epidermal sheets 4 days after HSV-1 skin infection, as in **Figure 6A**. Data from one experiment. (**C**) Analysis of IFN-γ^+^ *in vitro* activated gDT-II effector cells cultured in the presence of increasing numbers of CD45.2^−^ or CD45.2^+^MHC-II^hi^ cells from epidermal sheets 4 days after infection. Data from 1 (CD45.2^+^MHC-II^hi^ APCs) or 2 (CD45.2^−^ APCs) experiments. (**D**) Analysis of MHC-II expression by CD45.2^+^ and CD45.2^−^ cells isolated from epidermal sheets (Epi) 5 days after infection.
(TIF)

Figure S8 **Distinct regulation of IFN-γ production by CD4^+^ and CD8^+^ T cells during HSV-1 skin infection.** (**A**) Different distribution of IFN-γ^+^ CD4^+^ and CD8^+^ T_EFF_ cells during HSV-1 skin infection. IFN-γ^+^ CD4^+^ T_EFF_ cells are broadly distributed within infected skin and associated lymphoid tissues. By contrast, IFN-γ^+^ CD8^+^ T_EFF_ cells are strictly confined to epithelial skin regions harboring infectious virus, including the epidermis and hair follicles, and are absent from lymphoid tissues. (**B**) This distinct anatomical distribution of IFN-γ^+^ CD4^+^ and CD8^+^ T_EFF_ cells results from their different responsiveness towards stimulation by APCs. Irrespective of their infection status, MHC-II^+^ professional APCs, such as DCs, activate CD4^+^ T_EFF_ cells in skin epithelium, dermis and LNs, whereas nonprofessional APCs, such as keratinocytes or DETCs, fail to do so. By contrast, IFN-γ production by CD8^+^ T_EFF_ cells is triggered only by directly infected cells, the majority of which are nonprofessional epithelial APCs, such as keratinocytes and DETCs. Noninfected DCs in the dermis or draining LNs fail to elicit IFN-γ production by CD8^+^ T_EFF_ cells, even though they activate CD4^+^ T_EFF_ cells and initiate division and effector differentiation of naïve CD8^+^ T cells.
(TIF)

Movie S1 **Reduced velocity of CD8^+^ T_EFF_ cells in proximity to HSV-infected cells.** Wild-type mice were subjected to skin infection with HSV.CFP and received *in vitro* activated gBT-I cells 3 days post-infection. A representative 59 min time-lapse movie of infected skin taken 5 days post-infection. The second harmonic generation signal (2HG) marks the collagen-rich dermal layer. The time-lapse is shown at 8 frames per second. Movie is representative of 8 movies from *n* = 4 mice from 2 experiments. Scale bar, 30 µm.
(MP4)
