# Supplementary figures and images for: Distinct APC Subtypes Drive Spatially Segregated CD4+ and CD8+ T-Cell Effector Activity during Skin Infection with HSV-1
Source: PLoS Pathog. 2014 Aug 14;10(8):e1004303. doi: 10.1371/journal.ppat.1004303 (PMC4133397; doi:10.1371/journal.ppat.1004303)

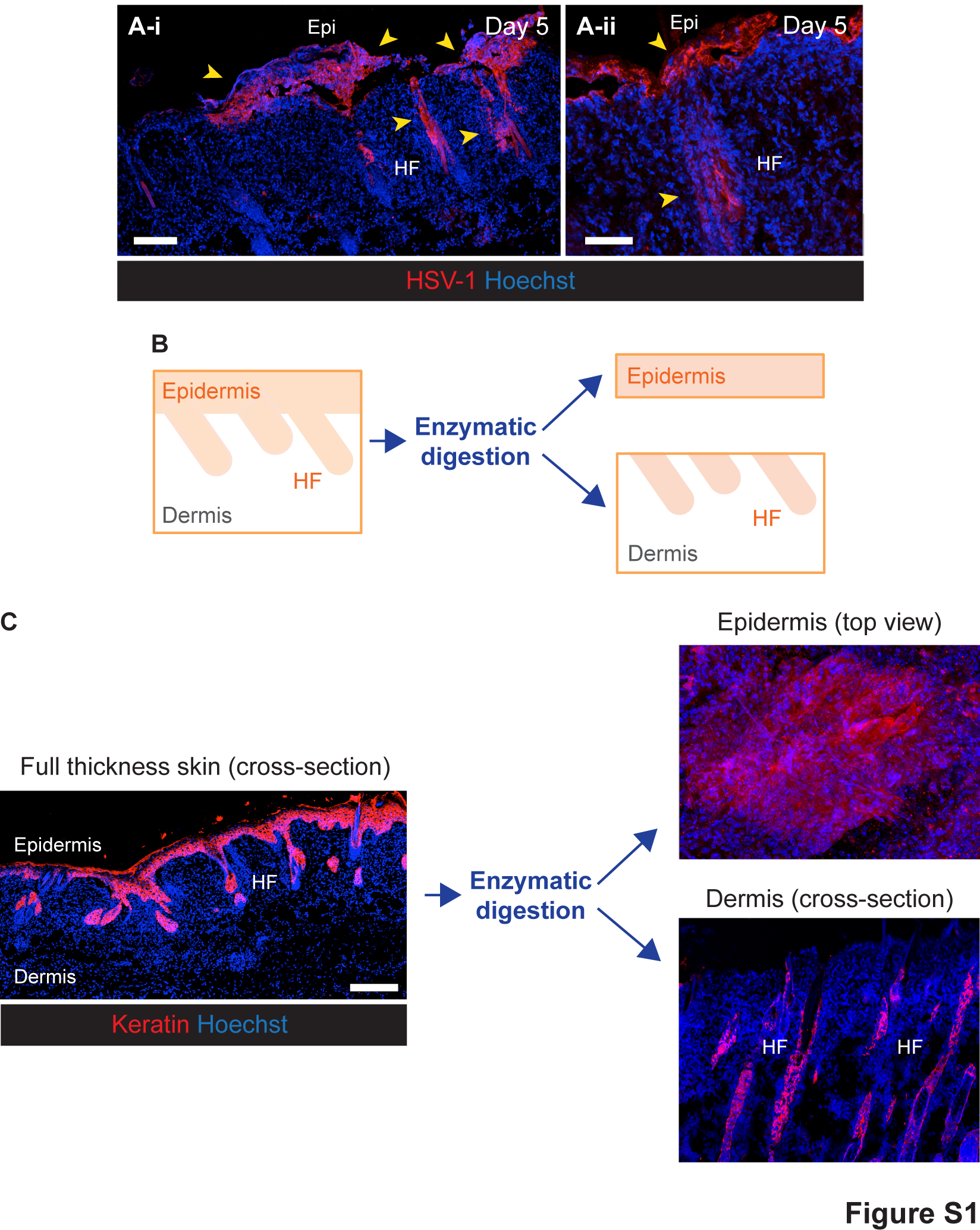

Supplement: Figure S1 — Topography of HSV-1 skin infection and preparation of epidermal sheets. (A) IFM analysis of skin stained with anti-HSV antibody 5 days after infection. Arrows indicate HSV-infected epithelial cells in the epidermis (Epi) and hair follicles (HF). Scale bars: A-i, 100 µm; A-ii, 50 µm. (B) Schematic diagram and (C) IFM analysis depicting the enzymatic and mechanical separation of epidermis and dermis. Note that the dermis preparation contains hair follicles (HF) of epithelial origin. Epithelial cells depicted by staining with anti-keratin antibody. Scale bar, 200 µm. (TIF) [file ppat.1004303.s001.tif]

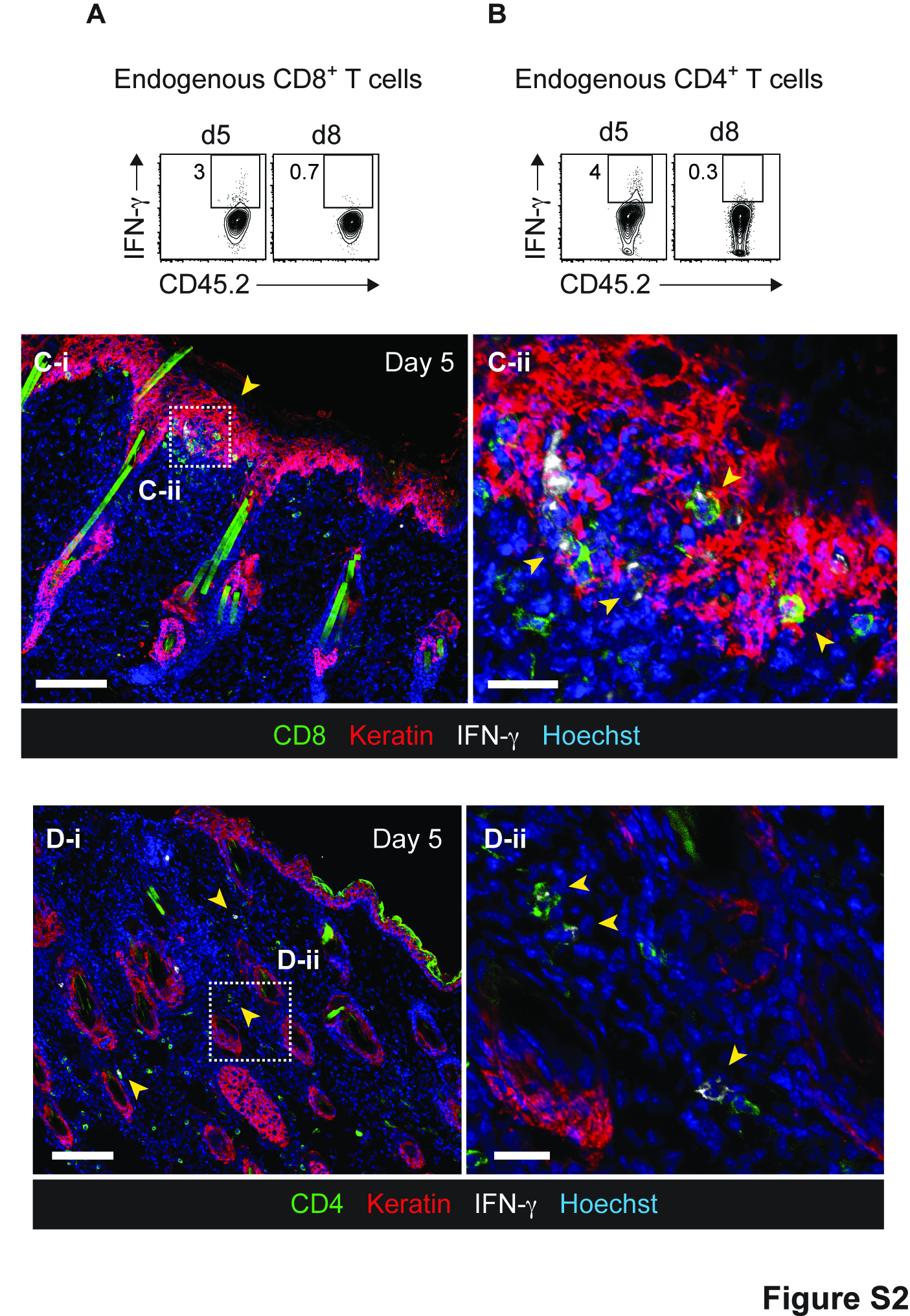

Supplement: Figure S2 — Distribution of endogenous IFN-γ+ TEFF cells in infected skin. (A,B) Mice were subjected to HSV-1 skin infection. Analysis of IFN-γ+ (A) CD8+ and (B) CD4+ T cells (collagenase digestion) 5 and 8 days post-infection. (C,D) IFM analysis of skin 5 days after infection stained with anti-HSV-1, -IFN-γ, -CD8 (C) or -CD4 (D) antibodies. Scale bars, C-i, 100 µm; C-ii, 20 µm; D-i, 100 µm, D-ii, 20 µm. Photos representative of n>5 sections from 2 mice/group. (TIF) [file ppat.1004303.s002.tif]

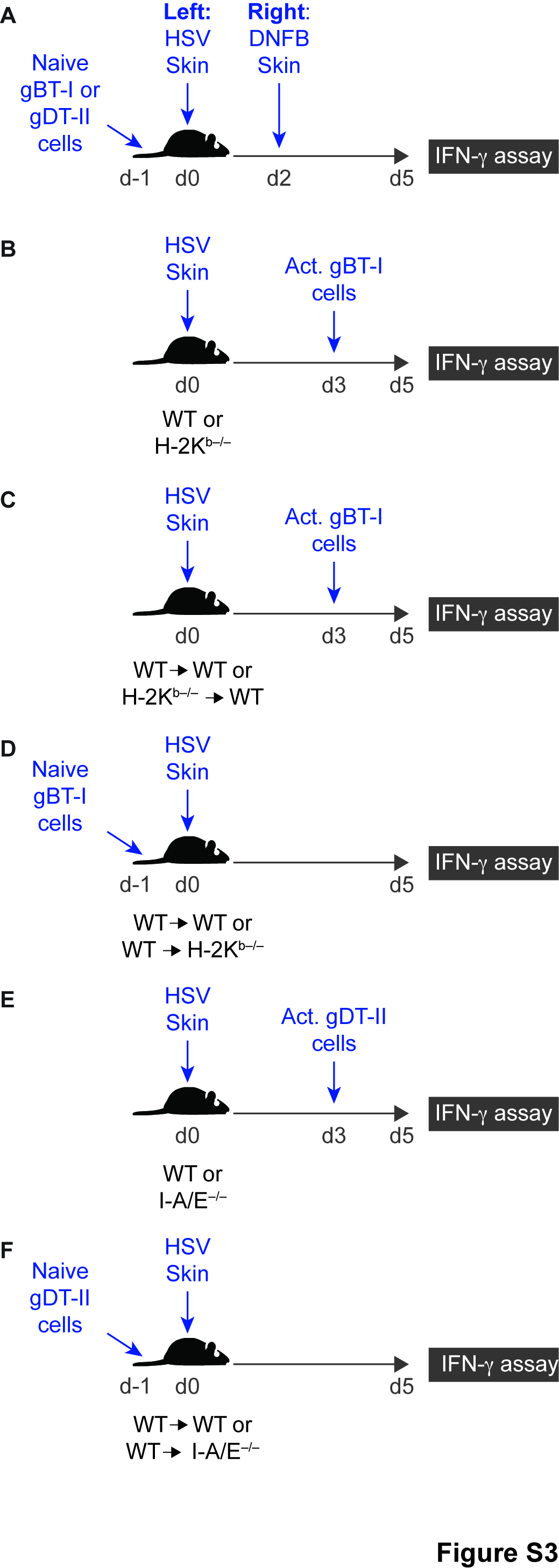

Supplement: Figure S3 — Experimental setups. (A) Experimental setup for Figure 3A . Wild-type mice received naïve gBT-I or gDT-II cells and 1 day later were subjected to HSV-1 skin infection on their left flank followed by skin treatment with DNFB on the right flank 2 days post-infection. Analysis of IFN-γ+ gBT-I and gDT-II cells from both flanks 5 days post-infection. (B,C) Experimental setup for Figure 3B–3D . Wild-type (WT) and H-2Kb−/− mice (B), or WT→WT and H-2Kb−/−→WT bone marrow chimeric (C) were subjected to HSV-1 skin infection and 3 days later received in vitro activated gBT-I effector cells. Analysis of IFN-γ+ gBT-I cells isolated from epidermal sheets 5 days post-infection. (D) Experimental setup for Figure 3E . WT→WT and WT→H-2Kb−/− mice received naïve gBT-I cells and 1 day later were subjected to infection. Analysis of IFN-γ+ gBT-I cells from epidermal sheets 5 days post-infection. (E) Experimental setup for Figure 3F and 3G . Wild-type (WT) and I-A/E−/− mice were subjected to HSV-1 skin infection and 3 days later received in vitro activated gDT-II cells. Analysis of IFN-γ+ gDT-II cells isolated from skin and axillary LNs 5 days post-infection. (F) Experimental setup for Figure 3H . WT→WT and WT→I-A/E−/− mice received naïve gDT-II cells and 1 day later were subjected to infection. Analysis of IFN-γ+ gDT-II cells from skin and axillary LNs 5 days post-infection. (TIF) [file ppat.1004303.s003.tif]

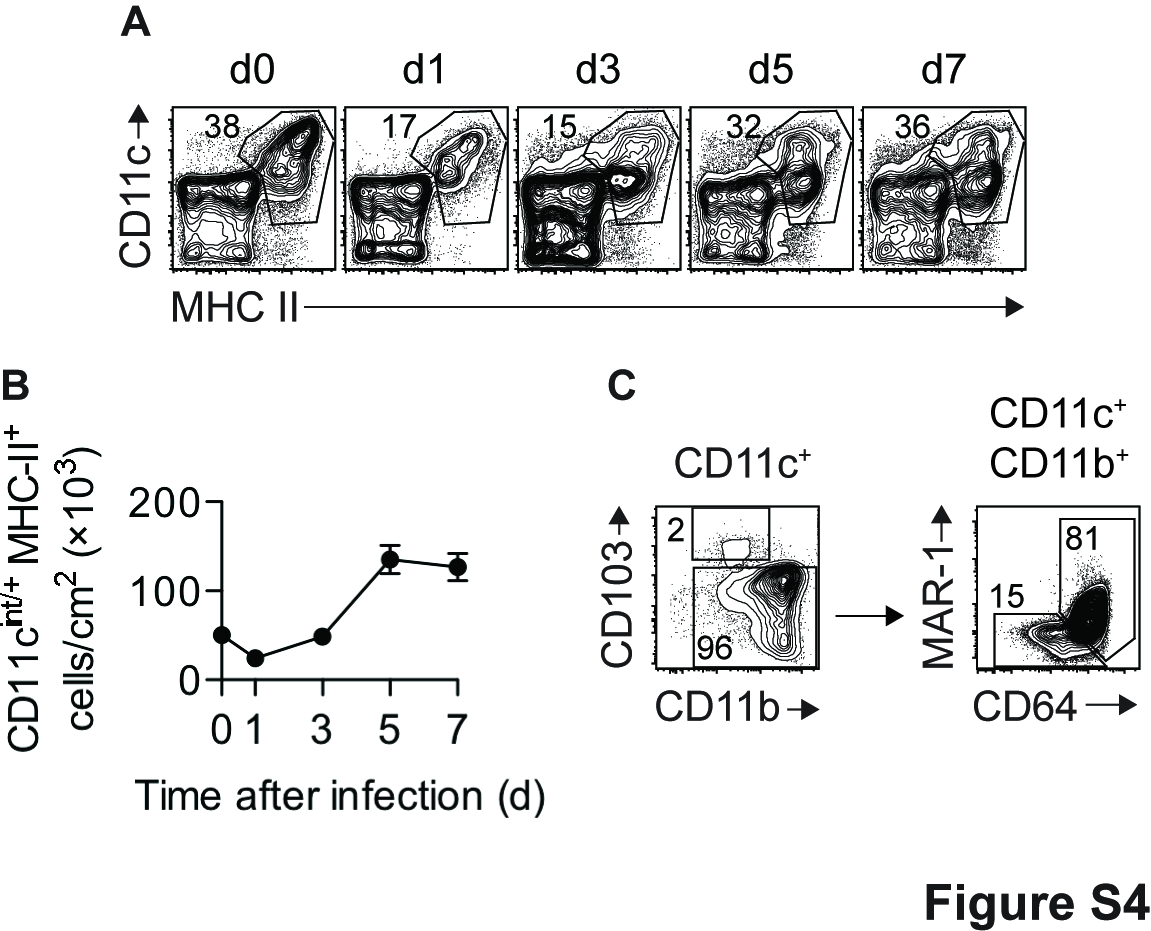

Supplement: Figure S4 — Infiltration of HSV-infected skin by CD11c+MHC-II+ APCs. (A–C) Mice were subjected to HSV-1 skin infection. Analysis of APCs from skin (collagenase digestion) at the indicated time points. (A) Plots gated on PI−CD45.2+ cells. (B) Enumeration of CD11cint/+MHC-II+ DCs. (C) Analysis of CD11b, CD103, CD64 and MAR-1 expression on DC populations gated as indicated. Data from n = 4–5 mice/per group. (TIF) [file ppat.1004303.s004.tif]

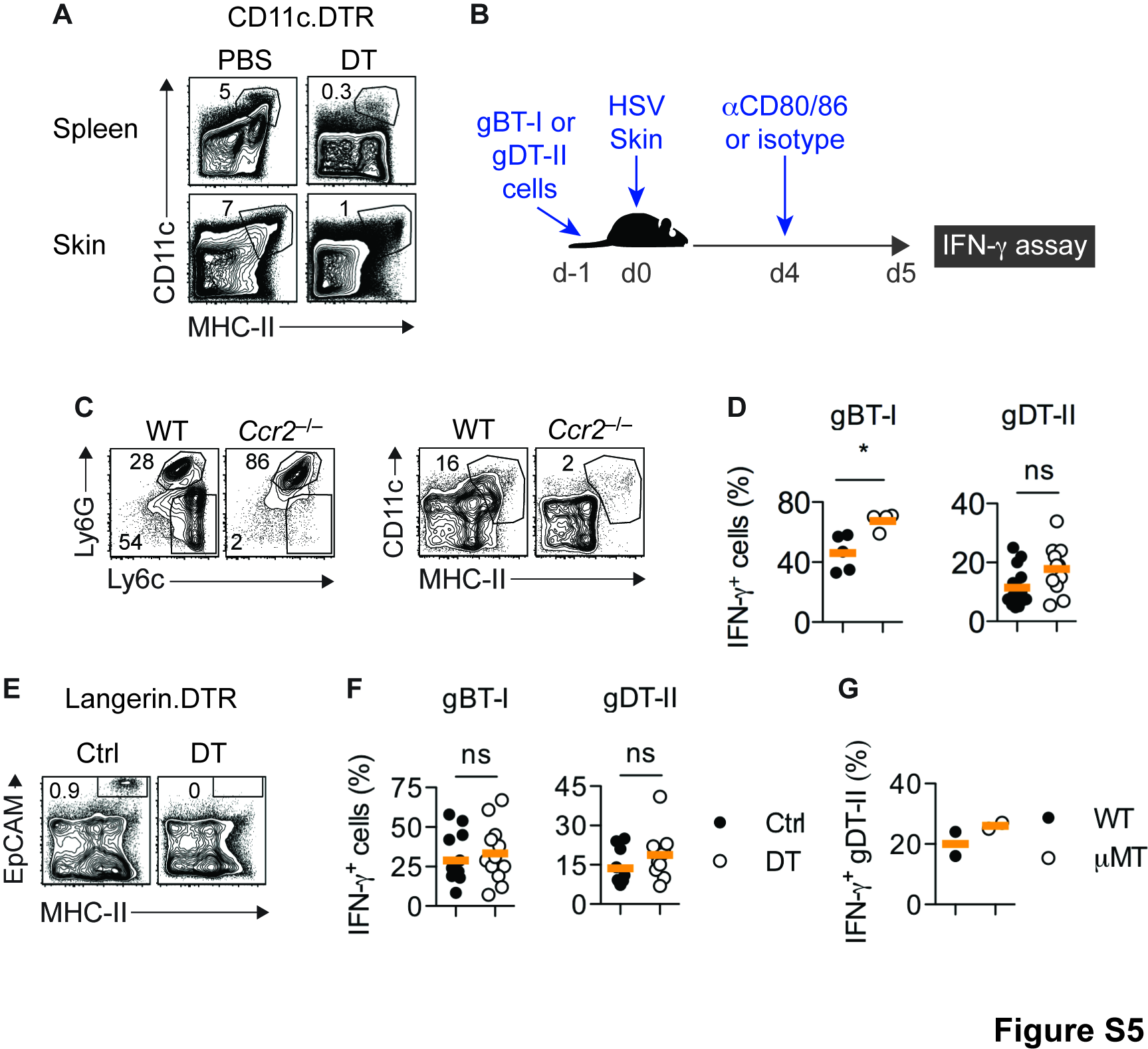

Supplement: Figure S5 — TEFF-cell activation in absence of specific APC subtypes. (A) Depletion of DCs in DT-treated CD11c.DTR mice. Representative plots, gated on CD45.2+PI− cells, show the proportion of CD11c+MHC-II+ DCs in spleen and skin of CD11c.DTR mice treated with PBS or DT according to Figures 4B and 4C . (B) Experimental setup for Figure 4D . Wild-type mice received naïve gBT-I or gDT-II cells prior to HSV-1 skin infection and were treated with blocking anti-CD80 and -CD86 or isotype control antibodies 4 days post-infection. Analysis of IFN-γ+ gBT-I and gDT-II cells in epidermal sheets (gBT-I, dispase digestion) or skin (gDT-II, collagenase digestion) and axillary LNs 5 days post-infection. (C,D) Wild-type (WT) or Ccr2 −/− mice received naïve gBT-I or gDT-II cells prior to HSV-1 skin infection. (C) Representative plots show the proportion of LyChiLy6G− monocytes and of CD11c+MHC-IIhi APCs in the skin of WT and Ccr2−/− mice 5 days post-infection. (D) Analysis of IFN-γ+ gBT-I (epidermis, dispase digestion) and gDT-II cells (dermis, collagenase digestion). *, P<0.05; ns, not significant by Mann Whitney test; n = 4–5 mice/group from 1 experiment (for gBT-I) and n = 11–16 mice/group from 4 experiments (for gDT-II). (E,F) Depletion of langerin+ cells in DT-treated Langerin.DTR mice. Langerin.DTR mice were subjected to HSV-1 skin infection and 4 days later treated with DT (500 ng) or PBS (Ctrl). Representative plots depict the proportion of Langerhans cells (MHC-IIhi EpCAM+) amongst PI−CD45.2+ cells in skin (collagenase digestion). (F) Mice received naïve gBT-I or gDT-II cells prior to infection. gBT-I-recipient mice were treated with DT (500 ng) or PBS (Ctrl) 4 days post-infection, and gDT-II-recipient mice were treated 2 days prior and 2 and 4 days after infection. Analysis of IFN-γ+ gBT-I cells in the epidermis (dispase digestion) or gDT-II cells in the skin (collagenase digestion). Ns, not significant by Mann Whitney test; n = 10–14 mice/group from 2–4 experiments. (G) Wild-type (WT [file ppat.1004303.s005.tif]

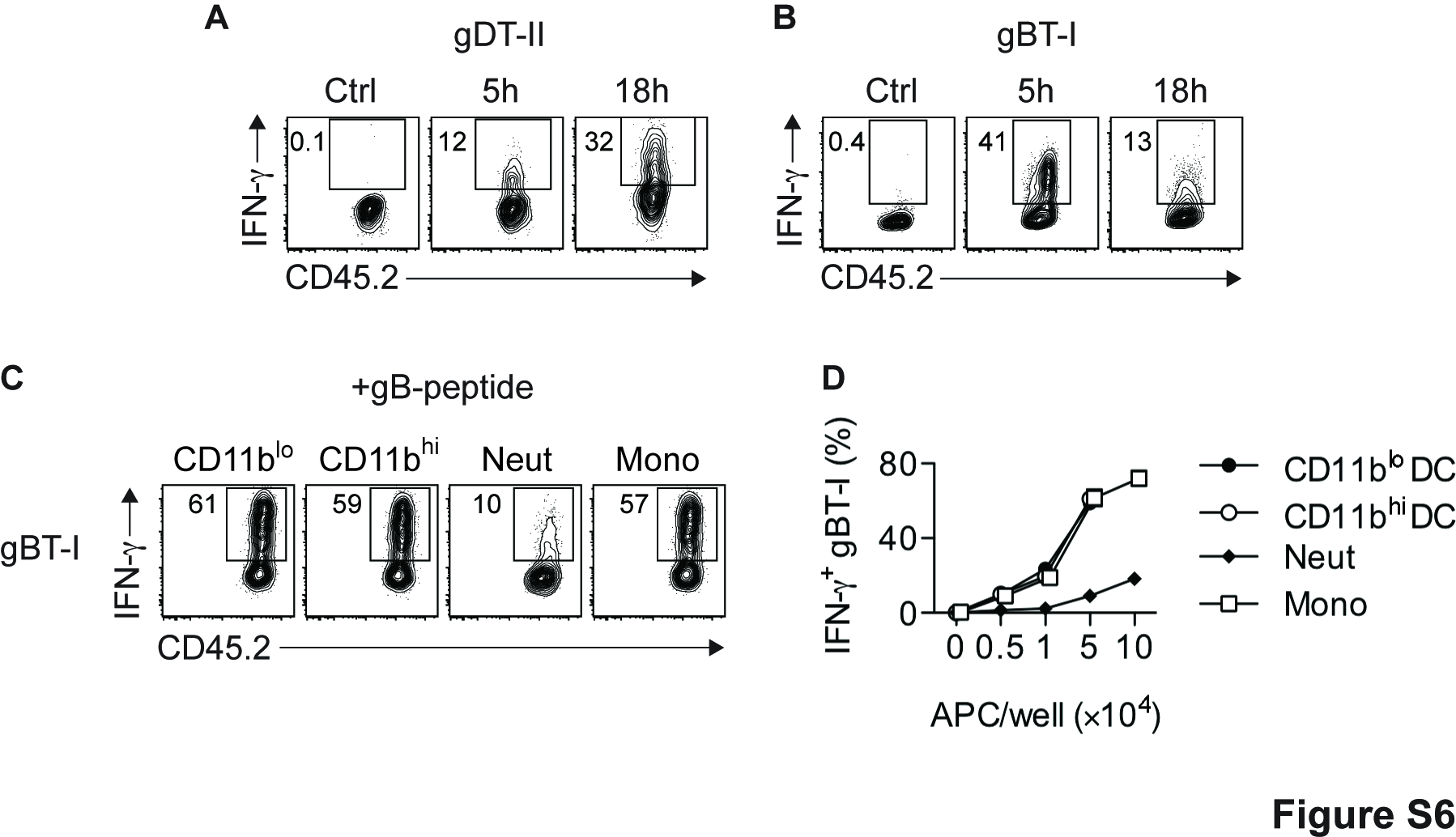

Supplement: Figure S6 — TEFF-cell IFN-γ production triggered by peptide-pulsed APCs. (A,B) Analysis of IFN-γ+ in vitro activated gBT-I and gDT-II effector cells cultured for 5–18 hours in the absence (Ctrl) or presence of gB498–505-peptide (gBT-I) or 1×10^5^ splenocytes and gD315–237-peptide (gDT-II). Representative plots gated on gBT-I and gDT-I cells, as indicated. (C,D) Mice were subjected to HSV-1 skin infection and 5 days post-infection APCs were isolated from skin (collagenase digestion), pulsed with 0.1 µg/mL gB498–505 peptide for 1 hour, and then sorted into CD11c+CD11blo and CD11c+CD11bhi DCs, CD11c−Ly6Cint neutrophils (Neut) and CD11c−Ly6Chi monocytes (Mono), as described in Figure 5A . (C,D) Analysis of IFN-γ+ in vitro activated gBT-I effector cells cultured for 5 hours in the presence of the indicated APC subsets (5×10^4^ each in C, increasing numbers as indicated in D). Data representative of (C) or pooled from (D) 2 experiments. (TIF) [file ppat.1004303.s006.tif]

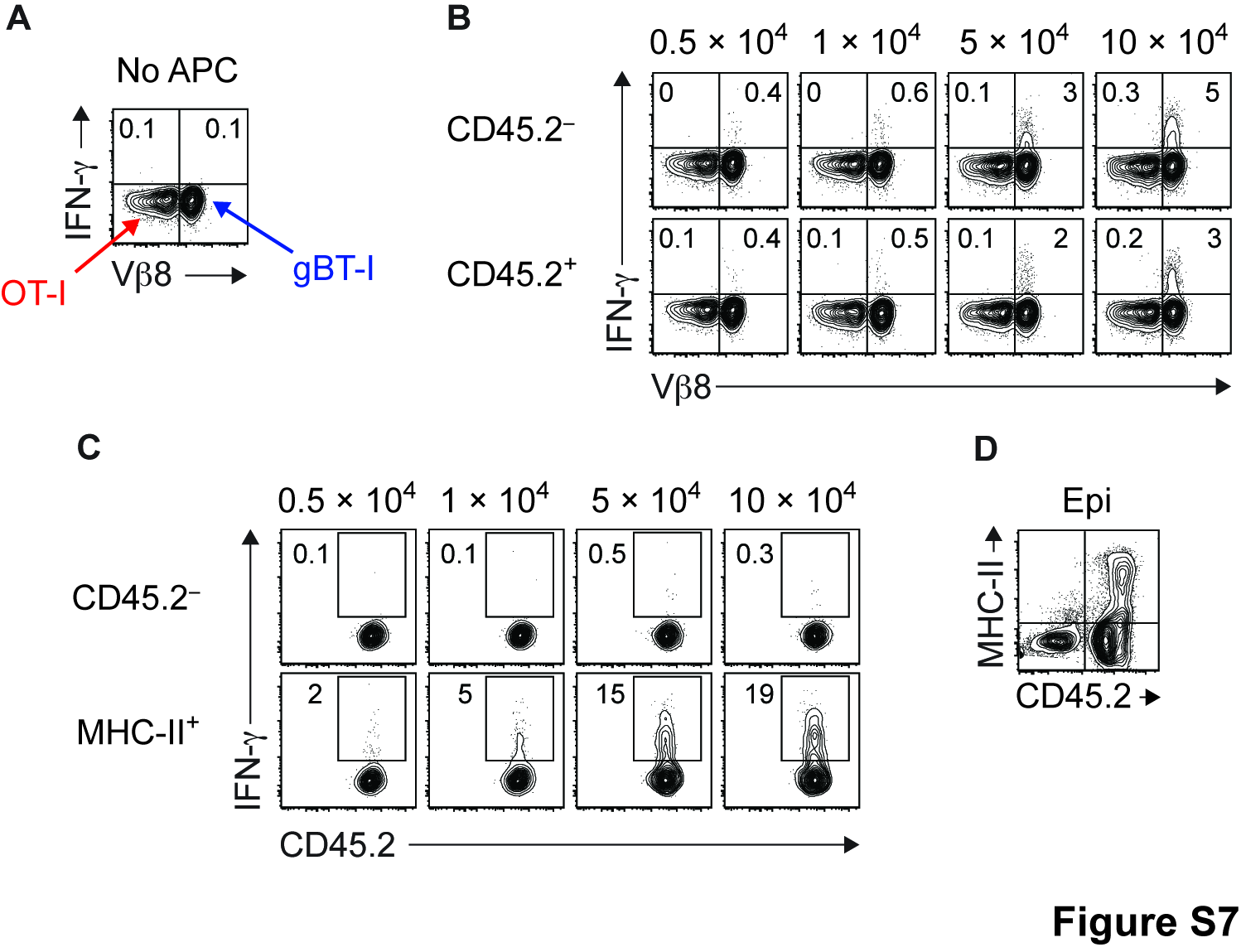

Supplement: Figure S7 — Distinct epidermal APC subsets trigger IFN-γ production by CD4+ and CD8+ TEFF cells. (A,B) Analysis of IFN-γ+ in vitro activated gBT-I (Vβ8+) and OT-I (Vβ8−) cells co-cultured in the absence (A) or presence of increasing numbers of CD45.2− or CD45.2+ cells (B) from epidermal sheets 4 days after HSV-1 skin infection, as in Figure 6A . Data from one experiment. (C) Analysis of IFN-γ+ in vitro activated gDT-II effector cells cultured in the presence of increasing numbers of CD45.2− or CD45.2+MHC-IIhi cells from epidermal sheets 4 days after infection. Data from 1 (CD45.2+MHC-IIhi APCs) or 2 (CD45.2− APCs) experiments. (D) Analysis of MHC-II expression by CD45.2+ and CD45.2− cells isolated from epidermal sheets (Epi) 5 days after infection. (TIF) [file ppat.1004303.s007.tif]

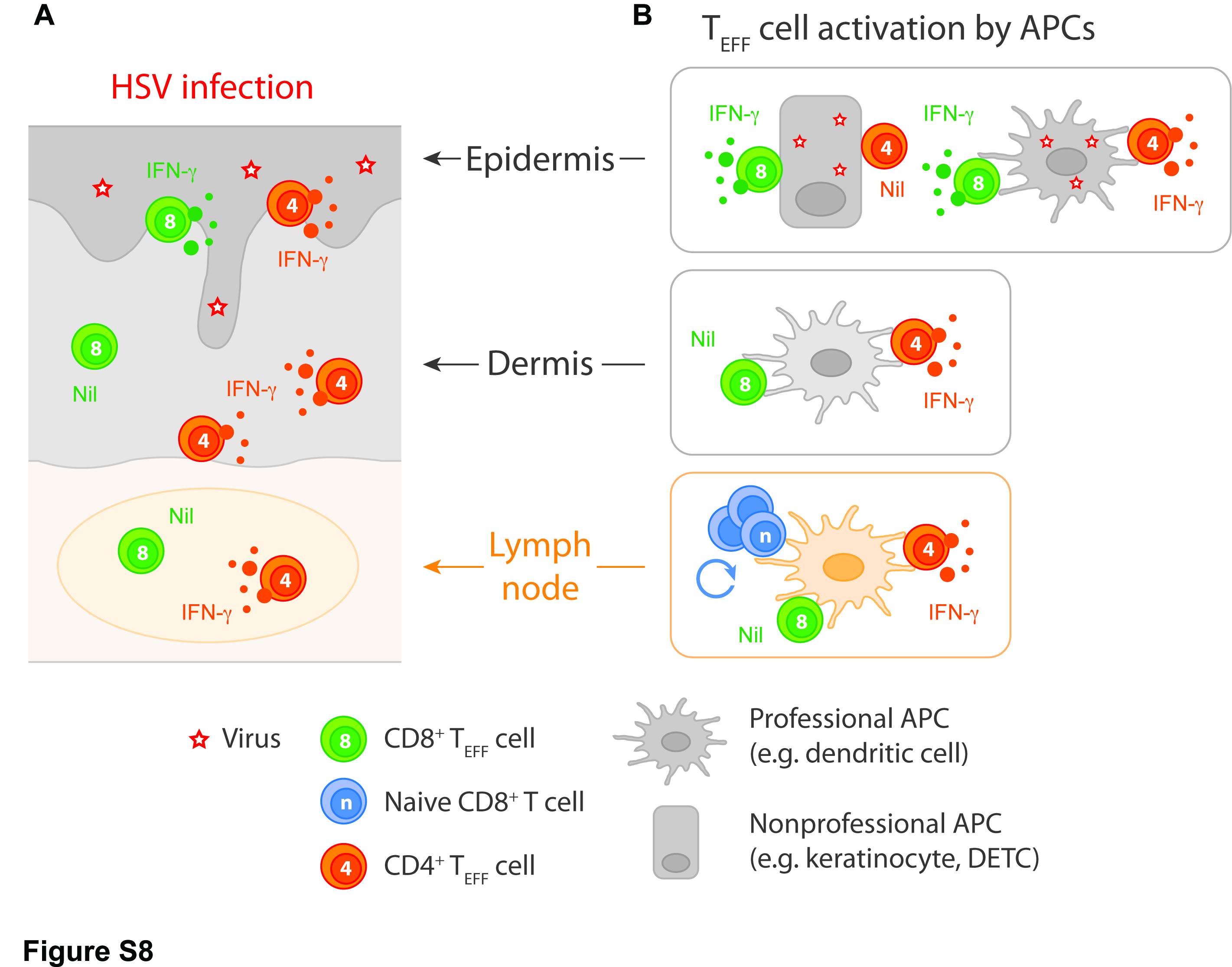

Supplement: Figure S8 — Distinct regulation of IFN-γ production by CD4+ and CD8+ T cells during HSV-1 skin infection. (A) Different distribution of IFN-γ+ CD4+ and CD8+ TEFF cells during HSV-1 skin infection. IFN-γ+ CD4+ TEFF cells are broadly distributed within infected skin and associated lymphoid tissues. By contrast, IFN-γ+ CD8+ TEFF cells are strictly confined to epithelial skin regions harboring infectious virus, including the epidermis and hair follicles, and are absent from lymphoid tissues. (B) This distinct anatomical distribution of IFN-γ+ CD4+ and CD8+ TEFF cells results from their different responsiveness towards stimulation by APCs. Irrespective of their infection status, MHC-II+ professional APCs, such as DCs, activate CD4+ TEFF cells in skin epithelium, dermis and LNs, whereas nonprofessional APCs, such as keratinocytes or DETCs, fail to do so. By contrast, IFN-γ production by CD8+ TEFF cells is triggered only by directly infected cells, the majority of which are nonprofessional epithelial APCs, such as keratinocytes and DETCs. Noninfected DCs in the dermis or draining LNs fail to elicit IFN-γ production by CD8+ TEFF cells, even though they activate CD4+ TEFF cells and initiate division and effector differentiation of naïve CD8+ T cells. (TIF) [file ppat.1004303.s008.tif]
